# The unfolded protein response and its activation by insulin in muscle are not altered by obesity or type 2 diabetes

**DOI:** 10.1042/CS20250639

**Published:** 2026-05-18

**Authors:** Rikke Kruse, Jonas Møller Kristensen, Birgitte Falbe Vind, Stine Juhl, Jacob Volmer Stidsen, Rugivan Sabaratnam, Kurt Højlund

**Affiliations:** 1 Steno Diabetes Center Odense, Odense University Hospital, DK-5000 Odense C, Denmark; 2 Department of Clinical Research, University of Southern Denmark, DK-5230 Odense M, Denmark; 3 The August Krogh Section for Molecular Physiology, Department of Nutrition, Exercise, and Sports, University of Copenhagen, DK-2100 Copenhagen, Denmark

**Keywords:** ER stress, Insulin, Obesity, Skeletal muscle, type 2 diabetes, Unfolded protein response

## Abstract

Insulin resistance in obesity and type 2 diabetes (T2D) is characterized by reduced insulin-stimulated glucose uptake, accumulation of triacylglycerol, mitochondrial dysfunction, and altered protein metabolism in skeletal muscle. This may involve disturbed endoplasmic reticulum (ER) homeostasis, leading to alterations in the unfolded protein response (UPR) and, hence, the protein folding capacity. Here, we investigated whether markers of UPR activity are elevated in skeletal muscle in obesity and T2D and to what extent insulin regulates these UPR markers. In a case–control design, we determined mRNA expression, protein abundance, and phosphorylation of key UPR markers in skeletal muscle biopsies obtained from patients with T2D, matched to glucose-tolerant individuals with obesity and lean individuals, before and after 4-h insulin infusion during a hyperinsulinemic-euglycemic clamp. The mRNA expression or protein abundance of GRP78, the canonical ER stress sensors (ATF6, PERK, and IRE-1α), several downstream UPR markers, and related markers of mitochondrial dynamics did not differ between groups. Insulin increased the mRNA expression of *ATF6*, *ERN1* (encoding IRE-1α), *XBP1*, *DDIT3* (encoding CHOP), and a marker of mitochondrial fission, *DNM1l* (encoding DRP1), as well as eIF2α Ser51 phosphorylation in skeletal muscle in all groups (all *P* <0.05), with no between-group differences. Our results demonstrate that markers of UPR activity are not elevated in skeletal muscle in obesity or T2D. Interestingly, insulin increases the expression of UPR markers and activates eIF2α, which is necessary for increasing the protein folding capacity of ER in muscle, and these responses are intact in obesity and T2D.

## Introduction

Obesity and type 2 diabetes (T2D) are characterized by insulin resistance in skeletal muscle. At the cellular level, this is characterized by a reduced insulin-stimulated glucose uptake and storage, accumulation of triacylglycerol, mitochondrial dysfunction, and altered protein metabolism [1–8]. Human skeletal muscle contains 50%–75% of all proteins in the body [9], and several cellular processes contribute to the regulation of the protein content in muscle, including autophagy and the ubiquitin-proteasome system [10]. The endoplasmic reticulum (ER) is a multifunctional organelle essential for cellular protein quality control, as it is responsible for protein synthesis, chaperone-mediated protein folding, post-translational modifications, and calcium storage. Disruption of the ER homeostasis is triggered by an imbalance between the ER capacity and the cellular demands for protein folding, which subsequently induces ER stress and a concomitant up-regulation of the unfolded protein response (UPR) [8,11,12]. The UPR attenuates protein synthesis and increases the capacity of the ER to handle unfolded proteins, thus restoring cellular proteostasis [8,11,13]. The activation of UPR is mediated through three main canonical ER stress sensors: activating transcription factor (ATF) 6, PKR-like ER kinase (PERK), and inositol-requiring element-1α (IRE-1α). These transmembrane proteins are inactive when bound to the ER chaperone glucose-regulated protein (GRP)78, also known as binding immunoglobulin protein (BiP). However, the accumulation of unfolded proteins in the ER lumen results in the dissociation of this chaperone and enables activation of eIF2α through PERK-mediated phosphorylation at Ser51, which ultimately leads to a global inhibition of protein translation [8,11,13]. Additionally, the abundance of several ER chaperones (including GRP78, GRP94, and protein disulfide isomerase (PDI)) will increase, thus improving the folding capacity in the ER [8,11,13]. As such, transient activation of UPR restores ER homeostasis, while chronic ER stress may contribute to skeletal muscle loss and insulin resistance [14–16]. Ultimately, continued and uncontrolled ER stress may lead to the induction of apoptosis through the activation of transcription factors ATF3 and C/EBP homologous protein (CHOP, encoded by the gene *DDIT3*) [17,18].

Obesity and T2D have consistently been associated with abnormalities in mitochondrial content and function in skeletal muscle [2,4,19,20], and some studies have furthermore suggested that T2D is also associated with dysregulated mitochondrial dynamics, as shown by reduced mRNA and protein content of the mitochondrial GTPase mitofusin 2 (MFN2) [21–23]. Mitochondrial dynamics is the coordinated interplay between mitochondrial fission mediated by OPA1 and FIS1 and mitochondrial fusion orchestrated by MFN1 and MFN2 [24]. In addition to its role in modulating mitochondrial fusion, MFN2 is found in ER-mitochondria contact sites and may be essential for the actual tethering of ER to mitochondria [25]. Interestingly, chemically induced ER stress leads to up-regulation of *MFN2* mRNA and protein levels in cultured mouse embryonic fibroblasts and cardiac myocytes [26], while liver-specific and whole-body *MFN2* knockout in mice impairs insulin signaling and increases the abundance of UPR markers in the liver and skeletal muscle, respectively [27].

Obesity and T2D are both characterized by hyperinsulinemia and dyslipidemia, and T2D also by hyperglycemia. These changes have all been associated with increased abundance of ER stress markers in various tissues in mice and humans [14,28–33]. In human adipose tissue, the expression of numerous markers of ER stress, including the phosphorylation of eIF2α, is increased with obesity and insulin resistance [30,31], while only a few ER stress markers, GRP78, XBP1, and CHOP, have been found increased in skeletal muscle of patients with T2D [14,33]. As such, the link between ER stress, obesity, and insulin resistance in human skeletal muscle remains to be further established to elucidate whether a potentially altered response to ER stress may contribute to disruption of proteostasis in skeletal muscle in obesity and T2D.

Finally, insulin administration has been shown to induce the UPR in both cultured 3T3-L1 adipocytes and in subcutaneous adipose tissue of individuals with overweight or obesity [34]. Insulin augments protein synthesis in skeletal muscle, leading to speculation that this process is accompanied by UPR induction to handle the increased load of misfolded proteins resulting from increased protein synthesis. However, whether insulin activates UPR markers in human skeletal muscle and whether this action of insulin is intact in insulin-resistant conditions such as obesity and T2D remain to be investigated.

Prompted by these findings, we hypothesized that obesity and T2D are associated with an increased abundance of UPR markers as a marker of chronic ER stress, as well as a blunted insulin-mediated activation of UPR markers in skeletal muscle. To test this, we investigated the basal levels of UPR markers and their regulation by physiological concentrations of insulin during a 4-h hyperinsulinemic-euglycemic clamp in the skeletal muscle of patients with T2D and well-matched, glucose-tolerant individuals with obesity and lean individuals.

## Research design and methods

### Study participants in the discovery cohort

The present study is a secondary analysis of muscle biopsy material collected in a previous project [1] and which has been used for other purposes in previous studies [35,36]. Similar to these secondary reports [35,36], two additional lean individuals were included in the present study. The present study included individuals with obesity and T2D (*n* = 10) that were matched for age and sex to glucose-tolerant individuals with obesity (*n* = 10) and lean volunteers (*n* = 12) as our discovery cohort (Table 1).

**Table 1:** Clinical and metabolic characteristics

Characteristics	Lean	Obese	T2D
*n* (female/male)	12 (6/6)	10 (4/6)	10 (6/4)
Age (years)	54.5 ± 1.3	55.4 ± 1.2	53.9 ± 1.6
BMI (kg/m^2^)	23.4 ± 0.5	31.1 ± 0.9***	29.8 ± 1.3***
Fat mass (kg)	17.6 ± 3.3	30.3 ± 1.9**	29.6 ± 1.3**
Fasting plasma glucose (mmol/l)	5.5 ± 0.1	5.8 ± 0.1	8.9 ± 0.7***^##^
Fasting serum insulin (pmol/l)	30 ± 4	40 ± 6	85 ± 16.3**^#^
HbA1c (%)	5.5 ± 0.1	5.3 ± 0.1	6.8 ± 0.4***^###^
Plasma triacylglycerols (mmol/l)	0.8 ± 0.1	1.0 ± 0.3	1.6 ± 0.2***^##^
GDR, basal (mg min^−1^ m^−2^)	76 ± 3	74 ± 6	73 ± 3
GDR, clamp (mg min^−1^ m^−2^)	380 ± 25	309 ± 24*	160 ± 26***^###^

The data are presented as mean ± SEM. *P <0.05, **P <0.01, and ***P <0.001 versus lean individuals, ^#^*P* <0.05, ^##^*P* <0.01, and ^###^*P* <0.001 versus obese individuals.

Abbreviations: BMI, body mass index; GDR, glucose disposal rate; T2D, type 2 diabetes.

People with T2D were GAD65-antibody negative and had no signs of diabetic neuropathy, nephropathy, retinopathy, or macrovascular complications. The patients with T2D were treated by diet alone or diet in combination with metformin, insulin, metformin and insulin, or rosiglitazone and sulfonylurea, as reported previously [1]. The individuals with obesity and lean control individuals had no family history of diabetes and a normal glucose tolerance, which was determined by an oral glucose tolerance test. None of the control individuals were treated with drugs known to affect glucose metabolism, and all participants were sedentary. Informed consent was obtained from all subjects before enrolment.

### Study participants in the validation cohort

A subset of 36 patients with T2D from the ‘specialist supervised individualized multifactorial treatment of new clinically diagnosed type 2 diabetes in general practice (IDA)’ study [37] and 12 individuals with normal glucose tolerance, matched on sex, BMI, age, and smoking status, were included as a validation cohort [38]. People with T2D were GAD65-antibody negative and were treated by diet alone (*n* = 2) or diet in combination with metformin (*n* = 24) or SGLT2 inhibitors (*n* = 4) or metformin and either DPP4 inhibitors (*n* = 4), SGLT2 inhibitors (*n* = 2), insulin (*n* = 3), or GLP-1 receptor agonists (*n* = 1). Informed consent was obtained from all individuals before participation.

### Study design

In both studies, the participants were instructed to refrain from strenuous physical activity for a period of 48 h before the experiment. One week prior to the examination, all oral glucose-, blood pressure-, and lipid-lowering drugs were withdrawn. Long-acting insulin was withdrawn the day before and rapid-acting insulin the night before the study. After an overnight fast, the participants underwent an euglycemic-hyperinsulinemic clamp to study the insulin-stimulated glucose disposal rates (GDR) as previously described [1,35–38], starting with a 2-h basal tracer (3-H^3^-glucose) equilibration period, which was followed by 4 h of insulin infusion at a rate of 40 mU m^−2^ min^−1^ with tracer glucose.

### Metabolic characterization of the discovery cohort

Plasma glucose, serum insulin, HbA1c, C-peptide, and triacylglycerols were measured in blood samples from the discovery cohort as previously described [39]. The fat mass percentage was determined by the bioimpedance method [39].

### Muscle biopsies

In both studies, the muscle biopsies were obtained from *m. vastus lateralis* before and after the 4-h insulin infusion period, using a modified Bergström needle with suction under local anesthesia (lidocaine). The biopsies were quickly blotted to remove any visible blood and connective tissue, then frozen in liquid nitrogen within 30 s, and thereafter stored at −80°C.

### RNA isolation and cDNA synthesis

For isolation of total RNA, 10–20 mg of muscle biopsy was homogenized in 1 ml TRI reagent (Sigma–Aldrich, St. Louis, MO) with 1.4 mm zirconium oxide beads using the PreCellys24 system at 5000 rpm for 2 × 20 s (Bertin Technologies, Montigny-Le-Bretonneux, France), and total RNA was extracted as previously described [35,36,40]. The RNA concentration was measured using a NanoDrop 1000 (Thermo Scientific, Waltham, MA). A total of 1500 ng of RNA from each muscle sample was treated with deoxyribonuclease I (Amplification Grade, Invitrogen, Carlsbad, CA) and reverse transcribed using a high-capacity cDNA reverse transcription kit according to the manufacturer’s instructions (Applied Biosystems, Foster City, CA).

### Quantitative real-time PCR analysis

The mRNA expression of a selected set of genes was measured by quantitative real-time PCR (qRT-PCR) performed on an ABI Prism 7900HT Sequence Detection System (Applied Biosystems, Foster City, CA) using TaqMan custom assays. The specific primer-probe pairs used are Hs99999907_m1 (*PPIA*), Hs99999904_m1 (*B2M*), Hs00232586_m1 (*ATF6*), Hs00358796_g1 (*DDIT3*), Hs00984006_m1 (*EIF2AK3*), Hs02856596_m1 (*XBP1U*), Hs00176385_m1 (*ERN1*), Hs00208382_m1 (*MFN2*), Hs01047018_m1 (*OPA1*), Hs00247147_m1 (*DNM1L*), and Hs00211420_m1 (*FIS1*), all from Applied Biosystems, Foster City, CA. All samples were run in technical triplicates, and the data analyzed using the qBasePlus Biogazelle software (Zwijnaarde, Belgium) [41,42]. The mRNA expression levels were normalized to the geometric mean of *PPIA* and *B2M*, which were found not to be affected by insulin, nor did they differ between the groups.

### Muscle lysate preparation

Frozen muscle biopsies were freeze-dried and dissected to remove visible fat, blood, and connective tissue before further analysis. For protein extraction, the freeze-dried and dissected muscles were homogenized (1:100 dw/vol) in ice-cold buffer (10% glycerol, 20 mM sodium pyrophosphate, 150 mM sodium chloride, 5 mM HEPES, 1% NP-40, 20 mM β-glycerophosphate, 10 mM sodium fluoride, 2 mM PMSF, 1 mM EDTA, 1 mM EGTA, 1 μg/ml aprotinin, 10 μg/ml leupeptin, 2 mM sodium orthovanadate, and 3 mM benzamidine; pH 7.5) by a tissue-lyzer (Tissue-Lyzer II, Qiagen Retch, Germany) for two times 1 min at 30 Hz. Muscle homogenates were rotated end-over-end at 4°C for 1 h, after which they were centrifuged (16,000×***g***/20 min). The supernatants were harvested as muscle lysate and stored at −80°C. The total protein content in the muscle lysates was determined by the bicinchoninic acid method (Pierce Chemical Company, Rockford, IL).

### SDS–PAGE and western blot analyses

Protein abundance and phosphorylation were measured by western blotting. Lysate proteins were separated by SDS–PAGE on self-cast Tris–HCl (6%–15%) PAGE gels and transferred to a PVDF membrane by semidry blotting (Immobilon Transfer Membranes; Millipore, Denmark). For each protein of interest, equal amounts of total lysate proteins were loaded for each sample on the gel. The western blots were performed using a balanced design in which samples from all experimental conditions were present on every gel. Internal control samples were included on each gel to correct for variations between gels, and furthermore, a standard curve was included to ensure that quantification of each protein investigated was within the linear range.

After transferring the proteins to the PVDF membrane, the membrane was blocked in washing buffer (10 mM Tris base, 150 mM NaCl, and 0.25% Tween 20, pH 7.4) containing low-fat milk protein (2%) or BSA (3%–5%) solution and afterward probed with primary antibodies and the appropriate secondary antibodies. The following antibodies were used: ATF3 (1:1000 in 3% BSA, #ab180842, Abcam, U.K.), GADD 153 R-20 (for detection of CHOP) (1:200 in 3% BSA, #sc-793, Santa Cruz Biotechnology, CA), eIF2α (D7D3) (1:1000 in 5% BSA, #5324, Cell Signaling Technology, MA), Phospho-eIF2α (Ser51) (D9G8) (1:1000 in 5% BSA, #5199, Cell Signaling Technology, MA), KDEL (10C3) (for detection of GRP94 (upper band) and GRP78/BiP (lower band)) (1:500 in 3% BSA, #ADI-SPA-827, Enzo Life Sciences, StressGen, NY), MFN2 (1:1000 in 3% BSA, #ab56889, Abcam, U.K.), PDI (C81H6) (1:1000 in 5% BSA, #3501, Cell Signaling Technology, MA), PERK (D11A8) (1:500 in 5% BSA, #5683, Cell Signaling Technology, MA), XBP-1U (M-186) (1:200 in 2% milk, #sc-7160, Santa Cruz Biotechnology, CA), XBP-1S (1 ug/ml in 2% milk, #ab37152, Abcam, U.K.). The secondary HRP-conjugated antibodies used were anti-rabbit (1:5000, #P0448, DakoCytomation, Denmark) and anti-mouse (1:5000, #P0161, DakoCytomation, Denmark).

Protein bands in the discovery study cohort were visualized using Fusion FX7 (Vilber Lourmat, Germany) after probing with enhanced chemiluminescence (Luminate Forte Western HRP Substrate, Millipore #WBLUF0500, MA or SuperSignal West Femto Maximum Sensitivity Substrate, Thermo Scientific #34096, IL). Bands were quantified using Science Lab MultiGauge version 3.0 (LifeScience Fujifilm, Japan). Protein bands in the validation cohort were quantified using ChemiDoc XRS+ (Bio-Rad Laboratories, Herlev, Denmark), and bands were quantified using ImageLab 6.1 (Bio-Rad Laboratories, Herlev, Denmark). All western blot images are included in Supplementary Figures S1–S11.

### Statistics

Statistical analyses were performed in SigmaPlot version 12.5 (Systat Software, CA, U.S.A.). The statistical evaluation of the clinical measures was performed using a one-way ANOVA, while the mRNA expression as well as protein abundances and phosphorylation were evaluated using a two-way ANOVA with repeated measures. Differences between groups were considered statistically significant when *P* <0.05. All data are expressed as means ± SEM.

## Results

### Clinical and metabolic characteristics

As reported previously [35], fasting plasma glucose, serum insulin and C-peptide, glycated hemoglobin (HbA1c), and plasma triacylglycerols were elevated in patients with T2D compared with individuals with obesity and lean control individuals (Table 1). Insulin-stimulated GDR (insulin sensitivity) was markedly reduced in patients with T2D compared with both individuals with obesity and lean control individuals (both *P* <0.001) and also to a minor extent in the obese group compared with the lean group (*P* <0.05). Similar differences in fasting plasma glucose, serum insulin, HbA1c, and insulin sensitivity between patients with T2D and weight-matched individuals were reported in the validation cohort [38].

### Insulin increases the transcription of UPR markers in human muscle

**Figure 1:**
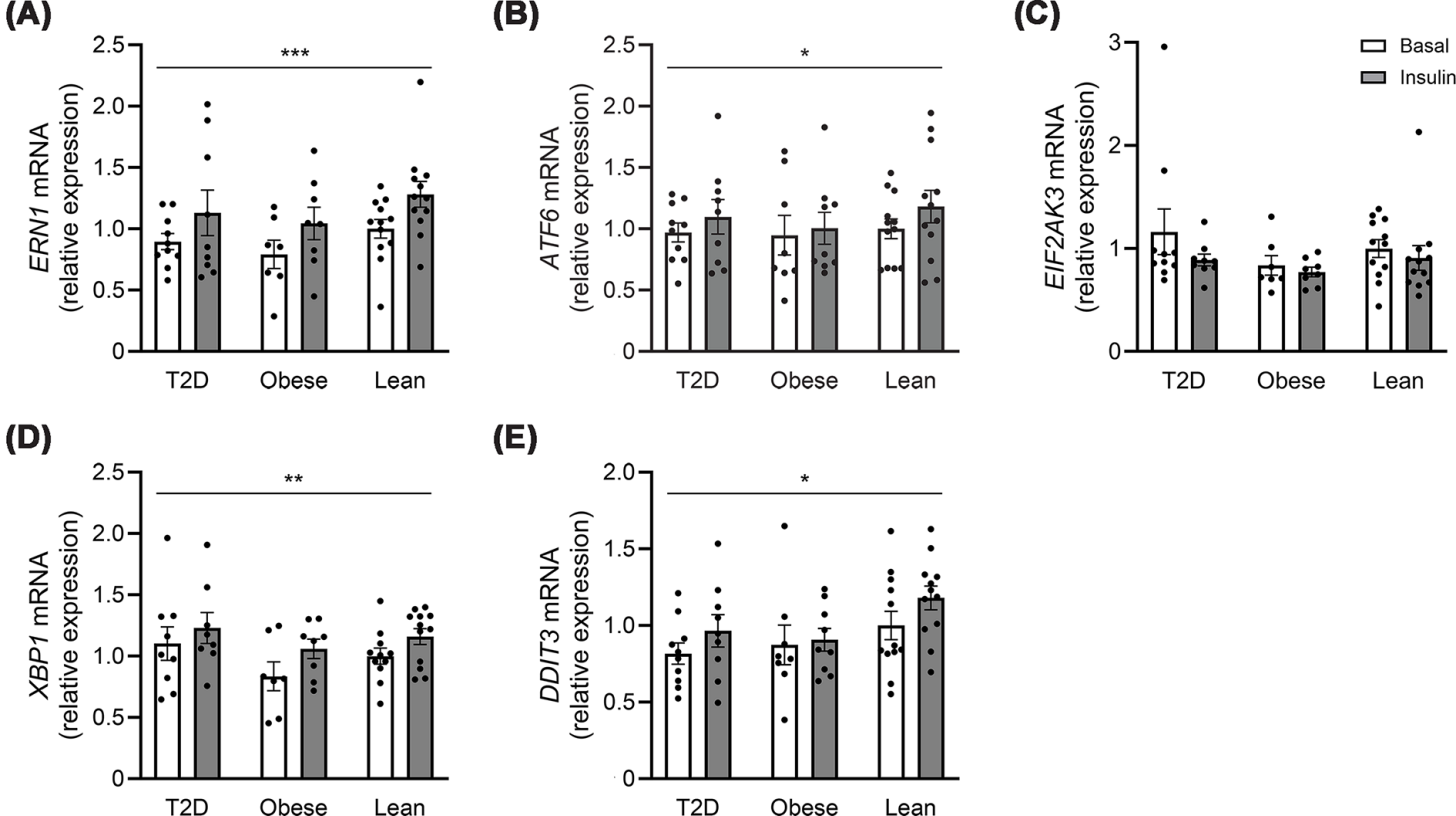
Transcriptional regulation of UPR markers in human skeletal muscle. mRNA expression of genes involved in UPR in the basal- (white bars) and insulin-stimulated (gray bars) state in skeletal muscle of lean individuals (*n* = 12) and individuals with obesity (*n* = 8–9) as well as in patients with T2D (*n* = 9–10) in the discovery cohort. (**A**) *ERN1* (encoding IRE-1α protein), (**B**) *ATF6*, (**C**) *EIF2AK3* (encoding PERK protein), (**D**) *XBP1U*, and (**E**) *DDIT3* (encoding CHOP protein). The data are presented as individual values as well as the mean ± SEM. *P <0.05, **P <0.01, and ***P <0.001 versus basal (main effect).

To investigate whether T2D and obesity are associated with increased UPR activation in human skeletal muscle, we assessed the transcript levels of components in all three branches of the UPR. The mRNA expression of the three upstream UPR modulators, *ERN1* (encoding IRE-1α), *ATF6*, and *EIF2AK3* (encoding PERK) did not differ between groups in the basal state (Figure 1A–C). Likewise, there were no differences between the groups in the basal expression of *XBP1* and *DDIT3* (encoding CHOP), which are downstream targets of IRE1 and PERK, respectively (Figure 1D,E). Four hours of insulin infusion increased the mRNA expression of *ERN1* (encoding IRE-1α, main effect *P* <0.001), *XBP1* (main effect *P* = 0.003), *ATF6* (main effect *P* = 0.018), and *DDIT3 (*encoding CHOP, main effect *P* = 0.017), but with no significant differences between the groups (Figure 1). The mRNA expression of *EIF2AK3* (encoding PERK) was not regulated by insulin (Figure 1C).

### Insulin activates eIF2α by Ser 51 phosphorylation in human muscle

**Figure 2:**
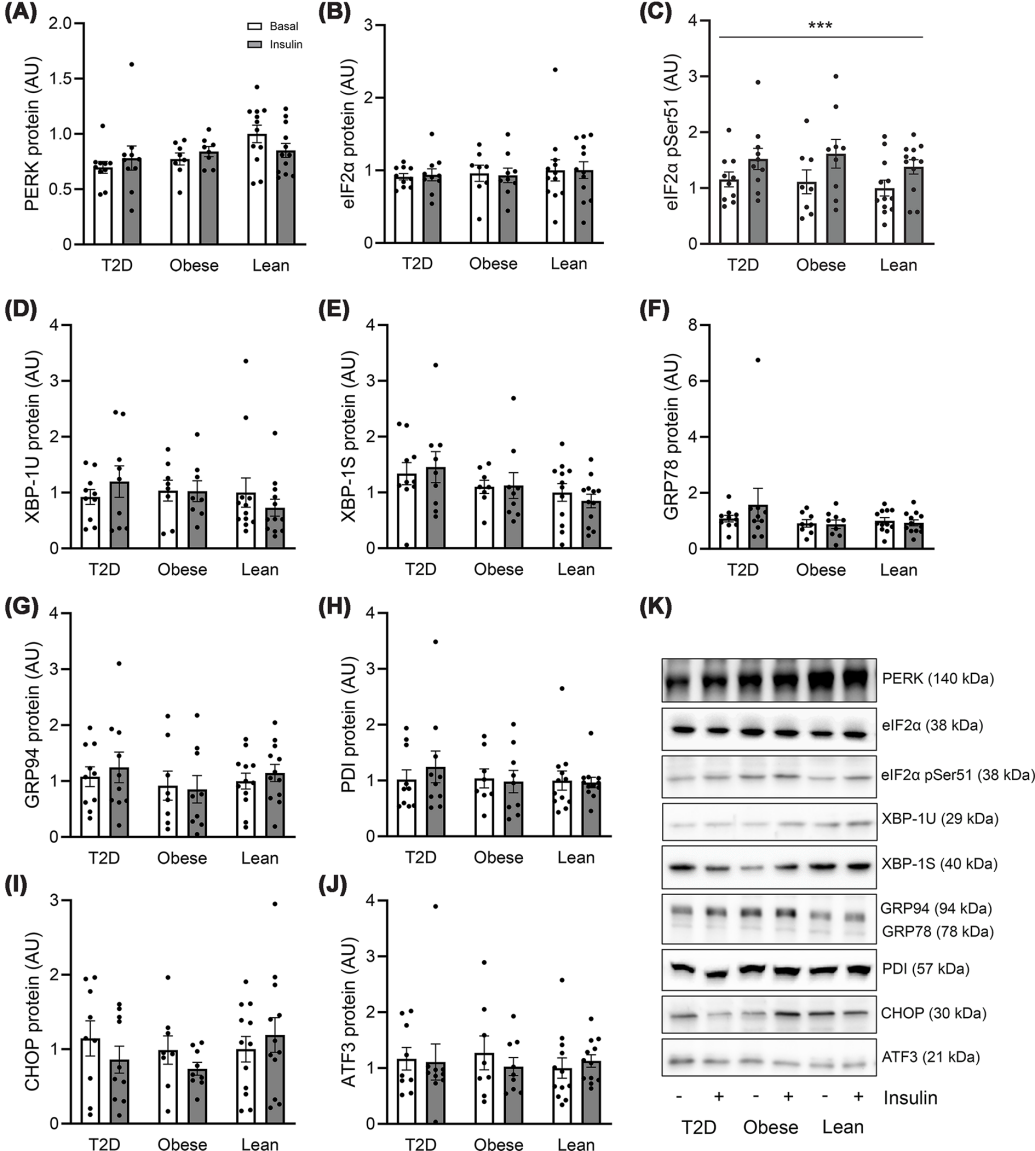
Protein abundance and phosphorylation of UPR markers in human skeletal muscle. Protein abundance and phosphorylation of markers in the UPR pathway in the basal- (white bars) and insulin-stimulated (gray bars) state in skeletal muscle of lean individuals (*n* = 12) and individuals with obesity (*n* = 8–9) as well as in patients with T2D (*n* = 9–10) in the discovery cohort. (**A**) PERK, (**B**) eIF2α, (**C**) eIF2α pSer51, (**D**) XBP-1U, (**E**) XBP-1S, (**F**) GRP78, (**G**) GRP94, (**H**) PDI, (**I**) CHOP, (**J**) ATF3, and (**K**) representative blots. The data are presented as individual values as well as the mean ± SEM. *P <0.05, **P <0.01, and ***P <0.001 versus basal (main effect). Uncropped, original blots are presented in Supplementary Figures S1–S9.

**Figure 3:**
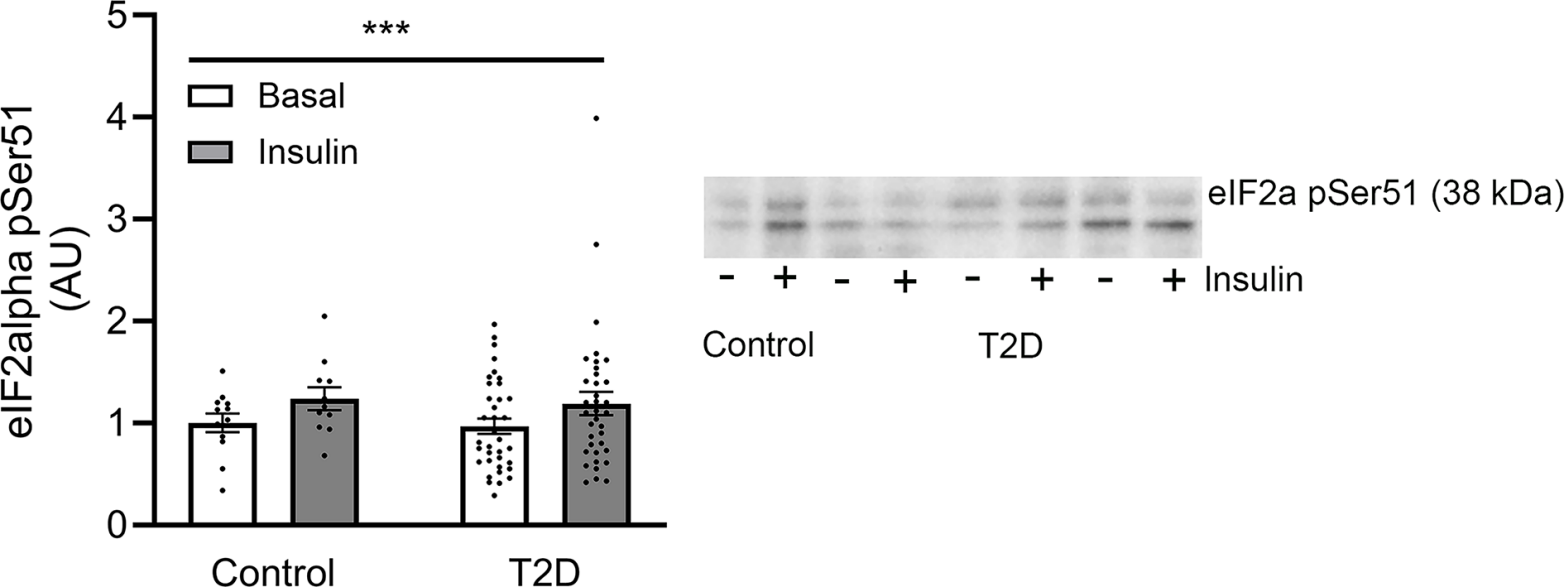
Phosphorylation of eIF2α in skeletal muscle of the validation cohort. Phosphorylation of eIF2α at pSer51 in the basal- (white bars) and insulin-stimulated (gray bars) state in skeletal muscle of patients with T2D (*n* = 36) and weight-matched glucose-tolerant individuals (*n* = 12) in the validation cohort. The data are presented as individual values as well as the mean ± SEM. ***P <0.001 versus basal (main effect). The uncropped, original blot is presented in Supplementary Figure S11.

Next, we investigated the protein abundance of PERK, eIF2α, XBP-1U (unspliced variant), XBP-1S (spliced and active variant), GRP78, GRP94, PDI, CHOP, and ATF3 as well as the activating phosphorylation of eIF2α at Ser51, which combined represents the three branches in ER stress sensing and UPR signaling. Overall, we did not identify any differences in protein abundance of these UPR markers between groups, nor did the protein abundance of the UPR markers change in response to physiological insulin concentrations for 4 h (Figure 2). However, insulin markedly (35%–44%) increased eIF2α phosphorylation at Ser51 in all groups of the discovery cohort (main effect, *P* <0.001) (Figure 2C), with no differences between groups. This effect of insulin was also evident when the phosphorylation of eIF2α at pSer51 was related to the eIF2α protein abundance (main effect *P* <0.001, Supplementary Figure S12), and still with no differences between groups. This ability of insulin to increase eIF2α phosphorylation at Ser51 was confirmed in the validation cohort consisting of 12 healthy control individuals and 36 patients with T2D (main effect, *P* = 0.001) with no significant differences between the groups (Figure 3).

### No effect of obesity and T2D on markers of mitochondrial dynamics

**Figure 4:**
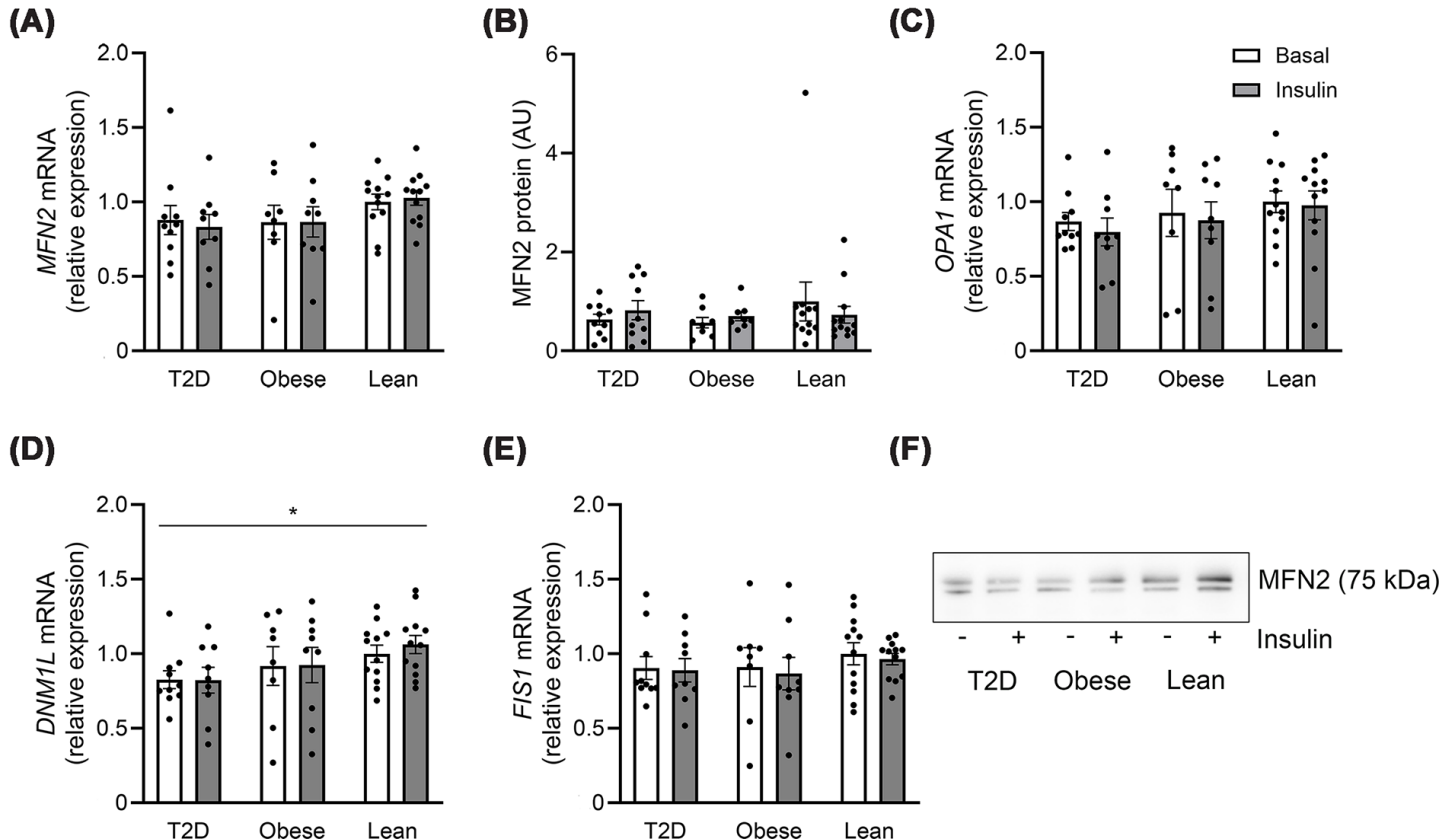
Gene expression and protein abundance of markers involved in mitochondrial dynamics in human skeletal muscle. Gene expression and protein abundance of markers involved in mitochondrial fission and fusion in the basal- (white bars) and insulin-stimulated (gray bars) state in skeletal muscle of lean individuals (*n* = 12) and individuals with obesity (*n* = 8–9) as well as in patients with T2D (*n* = 9–10) in the discovery cohort. (**A**) *MFN2* mRNA, (**B**) MFN2 protein, (**C**) *OPA1* mRNA, (**D**) *DNM1L* mRNA, (**E**) *FIS1* mRNA, and (**F**) representative western blots. The data are presented as individual values as well as the mean ± SEM. *P <0.05 versus basal (main effect). The uncropped, original blot is presented in Supplementary Figure S10.

The gene expression and protein content of MFN2, which is involved in ER-mitochondria tethering and mitochondria fusion, did not differ between groups in the basal state, nor was its gene expression or protein abundance influenced by insulin (Figure 4A,B). We also investigated other genes encoding proteins involved in the regulation of mitochondrial morphology, as they may influence mitochondrial homeostasis and thus potentially mitochondrial-specific UPR (UPR^mt^) [43]. Consistent with the *MFN2* data, we found that the expression of the fusion modulator *OPA1*, as well as the markers of mitochondrial fission, *DNM1L* (encoding dynamin-related protein 1 (DRP1) protein) and *FIS1* (encoding mitochondrial fission 1 protein), were equally expressed in the basal state in all three groups (Figure 4C–E). We observed a significant insulin-mediated increase in *DNM1L* mRNA expression (main effect, *P* = 0.017), with no significant differences between the groups (Figure 4D).

## Discussion

**Figure 5:**
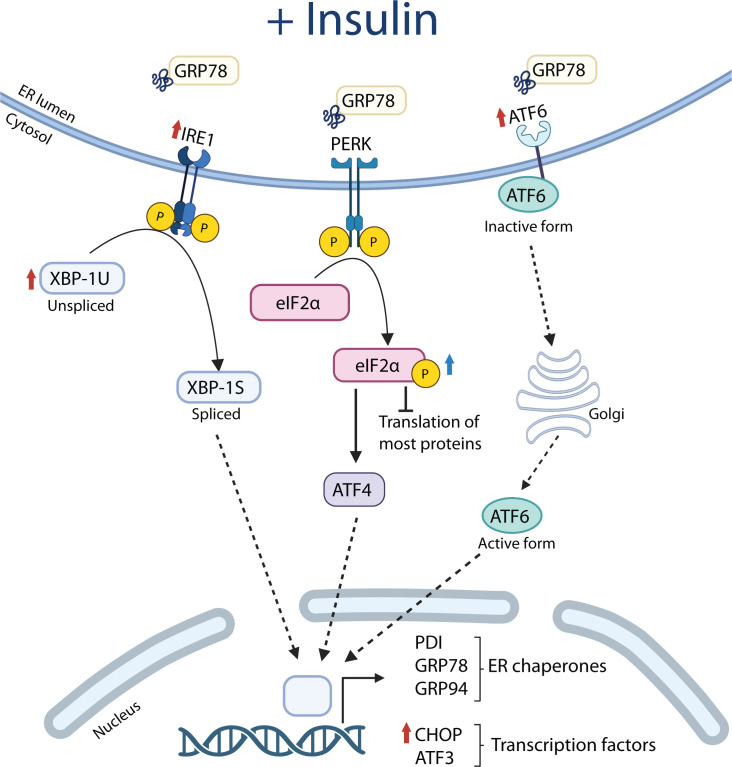
Graphical summary. Graphical summary of the significant changes mediated by insulin on selected markers of UPR in human skeletal muscle. A red arrow indicates a change in gene expression, whereas a blue arrow indicates a change in protein phosphorylation. Created in BioRender. Kruse, R. (2025). https://BioRender.com/kc8y5vs

In the present study, we examined whether obesity and T2D are associated with elevated markers of UPR activity and a blunted insulin-mediated activation of the UPR in human skeletal muscle. Here, we provide evidence that administration of insulin at physiological concentrations increases the expression of UPR markers in human skeletal muscle. This was evident by the insulin-mediated increase in UPR markers from all three branches of the canonical UPR pathway: *ATF6*, *ERN1* (encoding IRE-1α), *XBP1*, and *DDIT3* (encoding CHOP) mRNA, as well as the activation of eIF2α by Ser51 phosphorylation (Figure 5). Another main finding in the present study is that no baseline differences between groups were identified in any of the UPR markers investigated. Collectively, our findings suggest that UPR signaling is not chronically elevated in skeletal muscle in obesity or T2D and that the ability of insulin to induce UPR activation is necessary for increasing the protein folding capacity of ER in muscles and is preserved in obesity and T2D.

Obesity and T2D are characterized by reduced insulin-stimulated glucose uptake and metabolism, accumulation of triacylglycerol, reduced mitochondrial content and functional capacity, as well as altered protein metabolism in skeletal muscle [1–8]. The ER is an important organelle for cellular protein quality control, and there is evidence suggesting that T2D is associated with a dysfunctional ER in skeletal muscle, leukocytes, and adipose tissue [14,30–33]. However, only a few studies have systematically examined ER stress in T2D and obesity in human skeletal muscle [14,33,40]. In the present study, we did not observe indications of UPR activation in the skeletal muscle of patients with T2D when compared with either the glucose-tolerant individuals with obesity or lean control individuals following an overnight fast. This is somewhat inconsistent with previous findings, where an increased abundance of the UPR markers, GRP78, XBP1, and CHOP, has been reported in the skeletal muscle of patients with T2D compared with lean glucose-tolerant individuals [14,33]. It is, however, in line with our recent work, in which we did not identify differences in UPR at baseline or in response to an acute bout of exercise when comparing patients with T2D and weight-matched, glucose-tolerant men with obesity [40]. Thus, in contrast with our hypothesis, we found no evidence of increased UPR signaling and thus potentially chronic ER stress in muscle in obesity or T2D, suggesting that the protein quality control in skeletal muscle is to a major extent intact in these insulin-resistant conditions. Whether dysregulations in UPR and ER homeostasis exist in patients with more dysregulated T2D or morbid adiposity cannot be concluded from the present investigation. This highlights the need for further investigation of the canonical UPR pathways in relation to insulin resistance in obesity and T2D in a variety of different study designs and in a larger population.

To our knowledge, this is the first study identifying induction of UPR markers in human skeletal muscle in response to 4 h of physiological hyperinsulinemia. Our results are consistent with findings by Boden et al. in subcutaneous femoral adipose tissue of healthy humans, where supraphysiological insulin levels (∼1450 pmol/l) for 4 hours increased the mRNA expression of several UPR mediators, including *XBP1*, *GRP78*, and *ATF4* as well as the Ser51 phosphorylation of eIF2α [34]. Additionally, and in contrast with our findings, Boden *et al.* identified an insulin-mediated increase in the GRP78, ATF4, and ATF6 protein abundances in adipose tissue [34]. The study by Boden et al. also identified insulin-mediated changes in the protein levels of UPR markers when stimulating with lower insulin levels (480 pmol/l) during a prolonged clamp period (8 h versus 4 h in our study) [34]. These differences in study design may account for at least some of the discrepancies between the studies, as changes in protein abundance are more likely to be observed after 8 h rather than 4 h of insulin administration. Furthermore, it is likely that insulin influences ER stress and UPR differently in adipose tissue and skeletal muscle, respectively, and comparison of findings from different tissues should therefore be made with caution. Our results are supported by a recent proteomics study that included muscle samples from the validation cohort used in the present study [38]. Although this unbiased proteomic approach detected only 5 of the 11 UPR markers examined here and did not identify any phosphorylation sites on eIF2α, no significant differences in protein abundance between groups or regulation by insulin were observed for GRP78, eIF2α, GRP94, PDI, or DNM1L [38]. The inability to detect protein levels and phosphorylation sites for the key canonical ER stress sensors (ATF6, PERK, IRE1α) and eIF2α likely reflects the relatively low abundance of these UPR markers. These results underscore the value of hypothesis-driven, targeted investigations using classical methods or targeted proteomic approaches. Nevertheless, the insulin-mediated increase in *ATF6*, *ERN1*, *DDIT3*, and *XBP1* mRNA levels and phosphorylation of eIF2α observed in our study suggests that insulin induces UPR as a transient adaptive response to maintain ER homeostasis in human skeletal muscle. This is particularly relevant in a context where insulin stimulates protein translation and synthesis and thus the need for sufficient protein folding capacity.

Previous studies have suggested a connection between the mitochondrial GTPase MFN2, the ER, and the UPR [25–27,44] and also the existence of a mitochondrial-specific UPR has been identified [43]. Since obesity and T2D have consistently been associated with an impaired mitochondrial number and/or function in skeletal muscle [2,4,19], we examined the mRNA and protein levels of MFN2, as well as several other markers of the mitochondrial fusion and fission processes. We did, however, not identify any differences between groups in these markers of mitochondrial fusion and fission but observed a minor increase in mRNA levels of *DNM1L* in response to insulin. Therefore, in the present study, we do not find any evidence of either altered UPR activity, ER homeostasis, or mitochondrial dynamics in skeletal muscle in relation to obesity or T2D, despite several previous reports of mitochondrial dysfunction in muscle in obesity and T2D [2,4,19–23].

The observed insulin-mediated changes in the markers of UPR signaling could be explained by the anabolic effect of insulin, potentially leading to an enhanced protein synthesis rate and, therefore, a greater need for protein folding capacity. As such, insulin-mediated activation of the UPR could occur either because of transient ER stress as a result of an increased protein load or as a preventive measure where activation of UPR ensures an optimal balance between protein translation and protein folding, thus contributing to the maintenance of proteostasis. The design of our study does not allow us to discriminate between these two possibilities. Boden *et al.* substantiated the first possibility in human adipose tissue using proteomics to demonstrate increased abundance of 25 proteins and a higher degree of protein ubiquitination upon physiological insulin stimulation *in vivo*, hence suggesting increased proteasomal degradation of proteins [34]. However, whether these findings can be translated to skeletal muscle remains to be determined.

The scenario that insulin activates a part of the UPR proactively even before the development of ER stress is supported by studies reporting that insulin promotes the nuclear translocation of XBP-1S in cultured cells [45,46]. In fact, a combined proactive and reactive UPR activation may be of physiological advantage, i.e., following a meal where a rise in insulin and thus insulin-induced ER stress occurs [47]. Another mechanism by which insulin could transiently increase UPR markers may be through inhibition of autophagy. Acute hyperinsulinemia not only stimulates protein synthesis but also inhibits autophagy [35,48]. We have previously reported that insulin reduces the autophagic turnover (measured as a reduced LC3B-II/I protein ratio) in the participants with obesity and the lean individuals characterized in the present study [35]. Therefore, it is plausible that the apparent increase in UPR markers in response to insulin, at least in part, occurs as a consequence of reduced autophagic clearing of dysfunctional organelles and misfolded proteins, perhaps in addition to increased protein synthesis. The design of the present study and a lack of biopsy material do not allow us to further clarify mechanisms involved in the apparent insulin-mediated induction of UPR, and further studies are therefore needed to clarify this matter.

PERK is known to be activated by ER stress, and since most of the genes regulated by insulin in our study are upstream components of the UPR, we suggest that the observed insulin-mediated phosphorylation of eIF2α at Ser51 is mediated by PERK. However, other kinases such as PKR (protein kinase double-stranded RNA-dependent), GCN2 (general control non-derepressible-2), and HRI (heme-regulated inhibitor), as well as MARK2, have been demonstrated to phosphorylate eIF2α at Ser51 in vivo [49,50], and we cannot exclude that insulin activation of one of these kinases or, alternatively, inhibition of a phosphatase is responsible for this effect of insulin. Thus, further mechanistic studies are needed to establish the kinase or phosphatase involved.

The limitations of the present study include the small sample size, which was still sufficient to identify changes in insulin-stimulated GDR between groups. We may, therefore, have insufficient power to exclude small but potentially significant between-group differences in the gene expression and abundance and post-translational modification of the studied UPR markers. Furthermore, except for an overnight fast, we did not control or monitor the dietary intake prior to the studies for either the discovery cohort or the validation cohort. Finally, the observational nature of the study does not allow us to further investigate the mechanisms underlying our findings, including whether the observed changes are also associated with changes in the cellular localization, function, or activity of the UPR markers in skeletal muscle.

In summary, we demonstrate that insulin in physiological concentrations increases the expression of genes in all three branches of the canonical UPR pathway as well as the activation of eIF2α in human skeletal muscle and that these responses are intact in obesity and T2D (Figure 5). These findings suggest that the ability of insulin to increase the protein folding capacity is intact in these insulin-resistant conditions. In addition, our results provide evidence that markers of UPR activity in skeletal muscle are not chronically elevated in obesity or T2D. Overall, our findings indicate that these components of the ER homeostasis are not altered in relation to insulin resistance, although further studies in more dysregulated patients with T2D and individuals with morbid obesity are needed.

## Clinical perspectives

- Insulin resistance in obesity and T2D is characterized by reduced insulin-stimulated glucose uptake, accumulation of triacylglycerol, mitochondrial dysfunction, and altered protein metabolism in skeletal muscle. This may involve disturbed ER homeostasis, leading to alterations in the UPR and, hence, the protein folding capacity.
- Our results demonstrate that markers of UPR activity are not chronically elevated in skeletal muscle in obesity or T2D. Interestingly, insulin increases the expression of UPR markers and activates eIF2α, which is necessary for increasing the protein folding capacity of ER in muscle, and these responses are intact in obesity and T2D.
- Our findings suggest the existence of an insulin-mediated activation of UPR in human skeletal muscle.

## Supplementary Material

Supplementary Figures S1-S12

## Data Availability

All data and protocols are available upon request.

## Open Access

Open access for this article was enabled by the participation of the University of Copenhagen in an all-inclusive Read & Publish agreement with Portland Press and the Biochemical Society under an agreement with the Royal Danish Library.

## Abbreviations

ATF: activating transcription factor
BiP: binding immunoglobulin protein
CHOP: C/EBP homologous protein
ER: endoplasmic reticulum
ERAD: ER-associated protein degradation
GDR: glucose disposal rate
GRP: glucose-regulated protein
IRE-1α: inositol requiring element-1α
ISR: integrated stress response
MFN2: mitofusin 2
PDI: protein disulfide isomerase
PERK: PKR like ER kinase
qRT-PCR: quantitative real-time PCR
T2D: type 2 diabetes
UPR: unfolded protein response
