# Supplementary material for: The unfolded protein response and its activation by insulin in muscle are not altered by obesity or type 2 diabetes
Source: Clin Sci (Lond). 2026 May 18;140(6):961–73. doi: 10.1042/CS20250639 (PMC13199176; doi:10.1042/CS20250639)
Supplement: Supplementary Figures S1-S12 [file CS-2025-0639_supp.pdf]

Supplemental figure 1: Immuno blots of PERK (140 kDa) in the discovery cohort: a) Original image exported from software and b) original image inverted using ImageJ for improved visualisation. Amount of protein loaded (IC and samples): 30 ug. Amount of protein loaded in the standard curve: 15, 20, 30, and 60 ug. Exposure time: 20 sec.

The nature of the samples are indicated as follows: IC = Internal control, L- = Lean, basal, L+ = Lean, insulin, O- = Obese, basal, O+ = Obese, insulin, T- = T2D, basal, and T+ = T2D, insulin

The samples were run on six gels, which were cut around the band of interest and blotted onto a single membrane to avoid blotting-related differences across the study. The band indicted by the arrow was quantified. A standard curve made from a pooled samples was included on one of the gels as indicated.

a) Original image exported from software

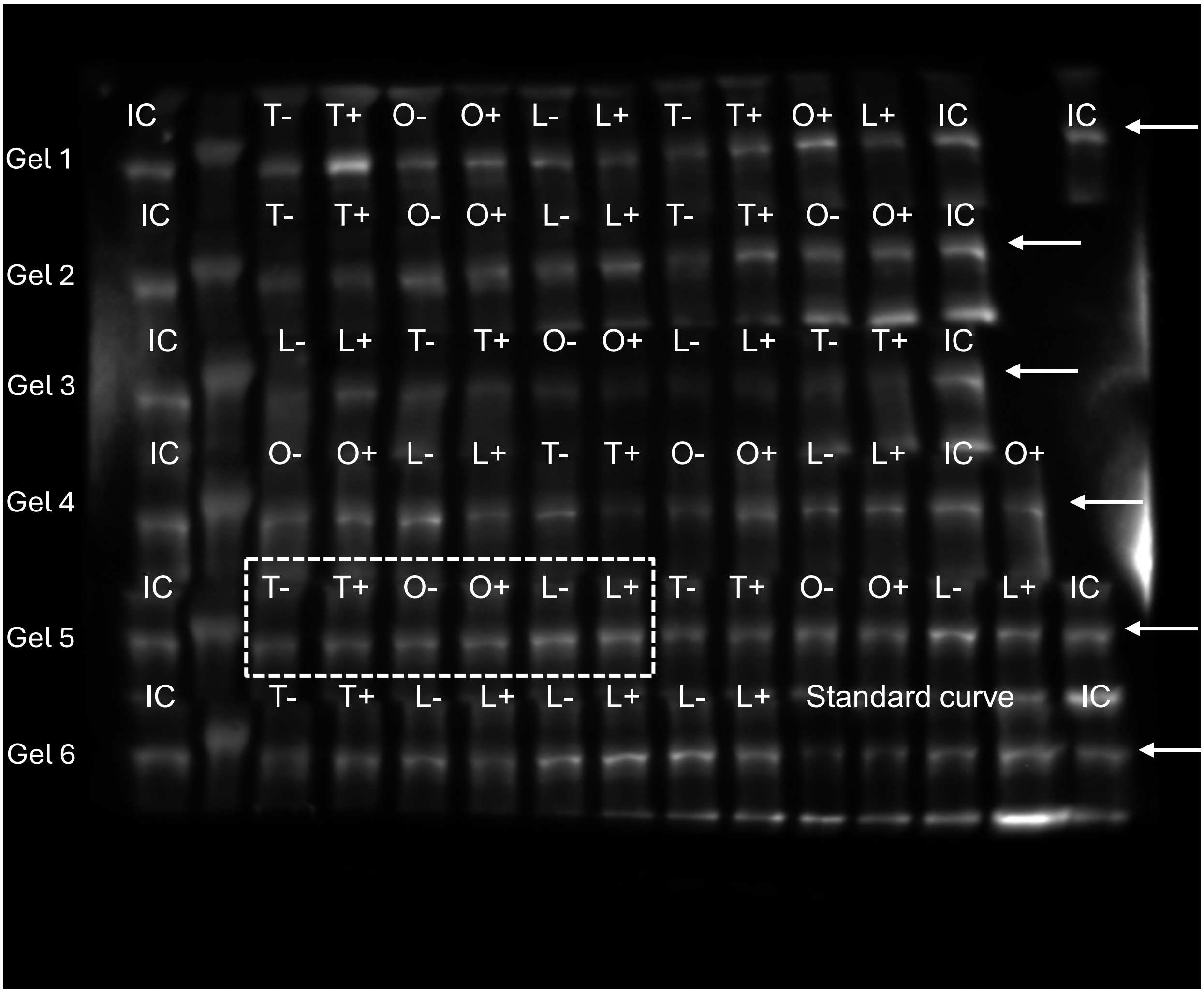

b) Original image inverted using ImageJ for improved visualisation

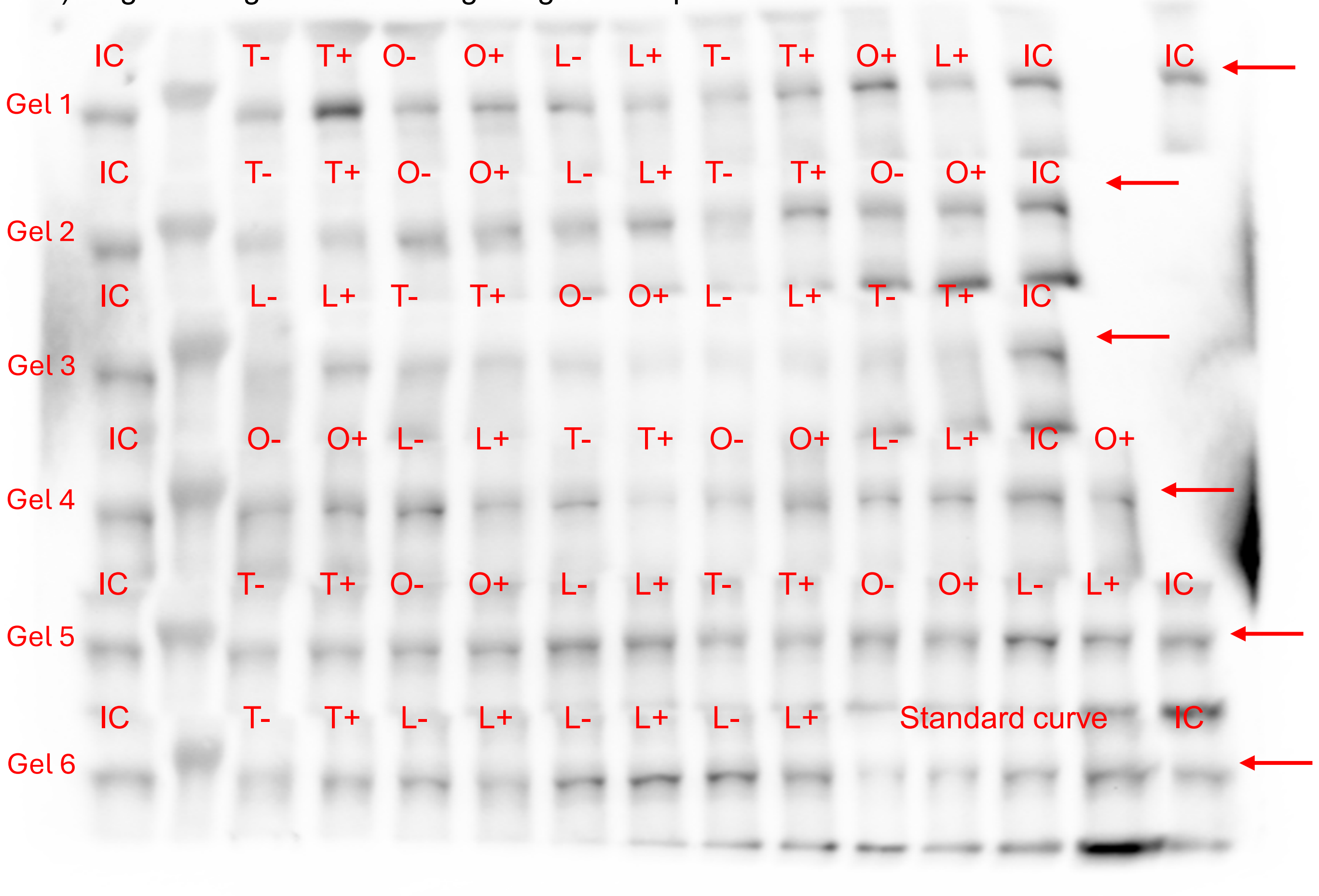

Supplemental figure 2: Immuno blots of eIF2α protein (38 kDa) in the discovery cohort: a) Original image exported from software and b) original image inverted using ImageJ for improved visualisation. Amount of protein loaded (IC and samples):6 ug. Amount of protein loaded in the standard curve: 3, 6, 9, and 12 ug. Exposure time: 20 sec.

The nature of the samples are indicated as follows: IC = Internal control, L- = Lean, basal, L+ = Lean, insulin, O- = Obese, basal, O+ = Obese, insulin, T- = T2D, basal, and T+ = T2D, insulin

The samples were run on six gels, which were cut around the band of interest and blotted onto a single membrane to avoid blotting-related differences across the study. The band indicted by the arorow was quantified. A standard curve made from a pooled samples was included on one of the gels as indicated.

a) Original image exported from software

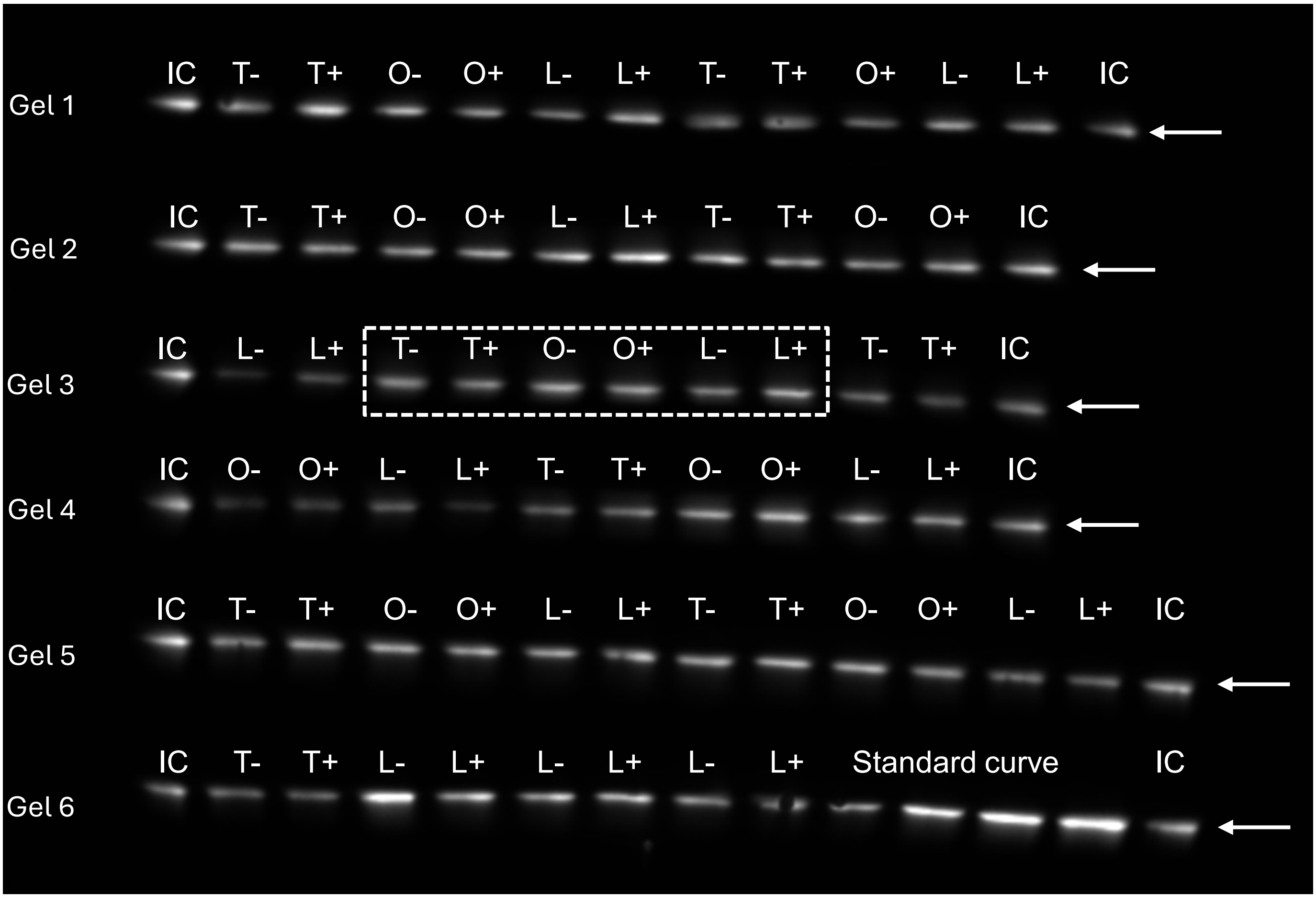

b) Original image inverted using ImageJ for improve visualisation

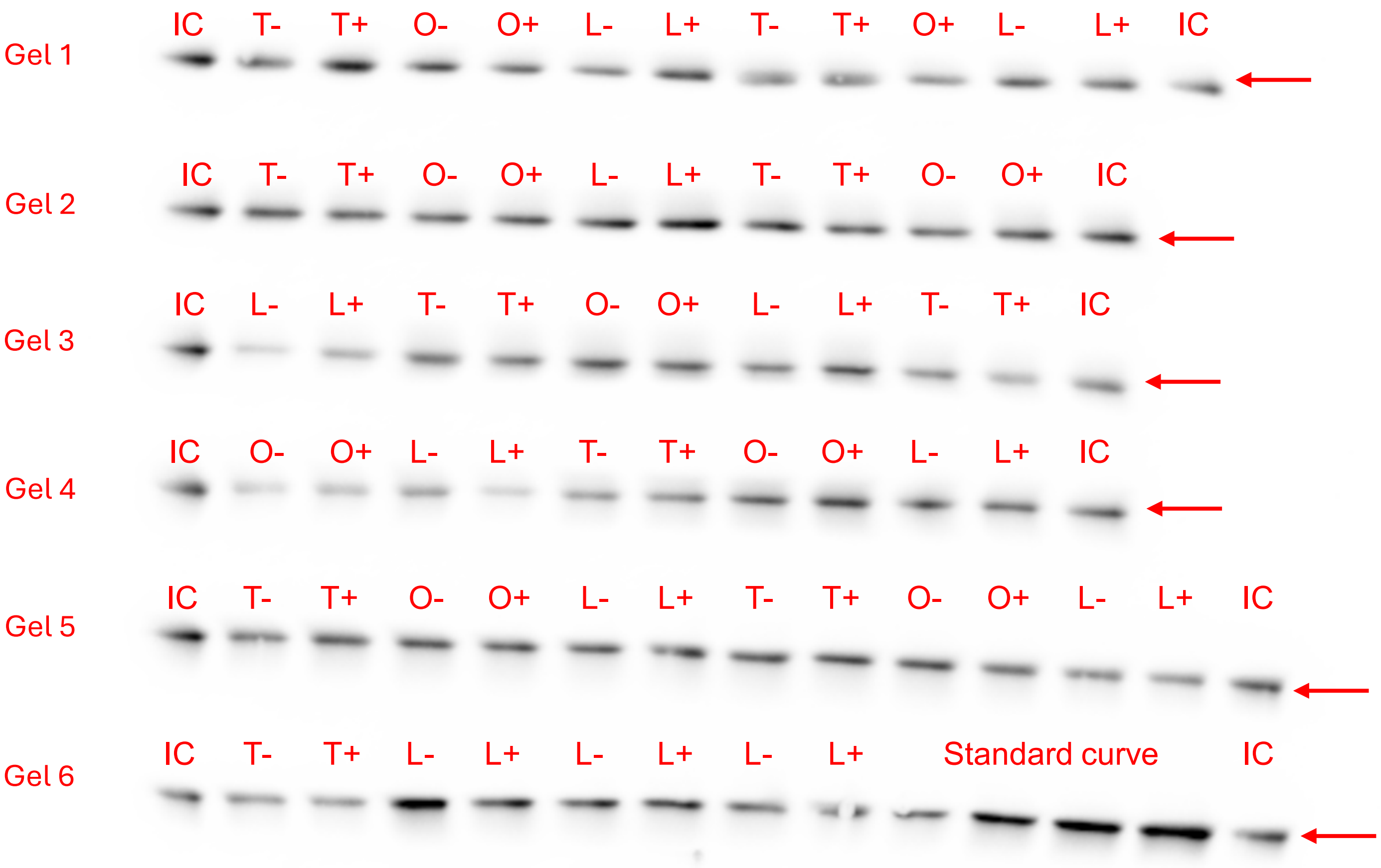

Supplemental figure 3: Immuno blots of eIF2α pSer51 (38 kDa) in the discovery cohort: a) Original image exported from software and b) original image inverted using ImageJ for improved visualisation. Amount of protein loaded (IC and samples):30 ug. Amount of protein loaded in the standard curve: 10, 20, 30, and 40 ug. Exposure time: 20 sec.

The nature of the samples are indicated as follows: IC = Internal control, L- = Lean, basal, L+ = Lean, insulin, O- = Obese, basal, O+ = Obese, insulin, T- = T2D, basal, and T+ = T2D, insulin

The samples were run on six gels, which were cut around the band of interest and blotted onto a single membrane to avoid blotting-related differences across the study. The band indicted by the arorow was quantified. A standard curve made from a pooled samples was included on one of the gels as indicated.

a) Original image exported from software

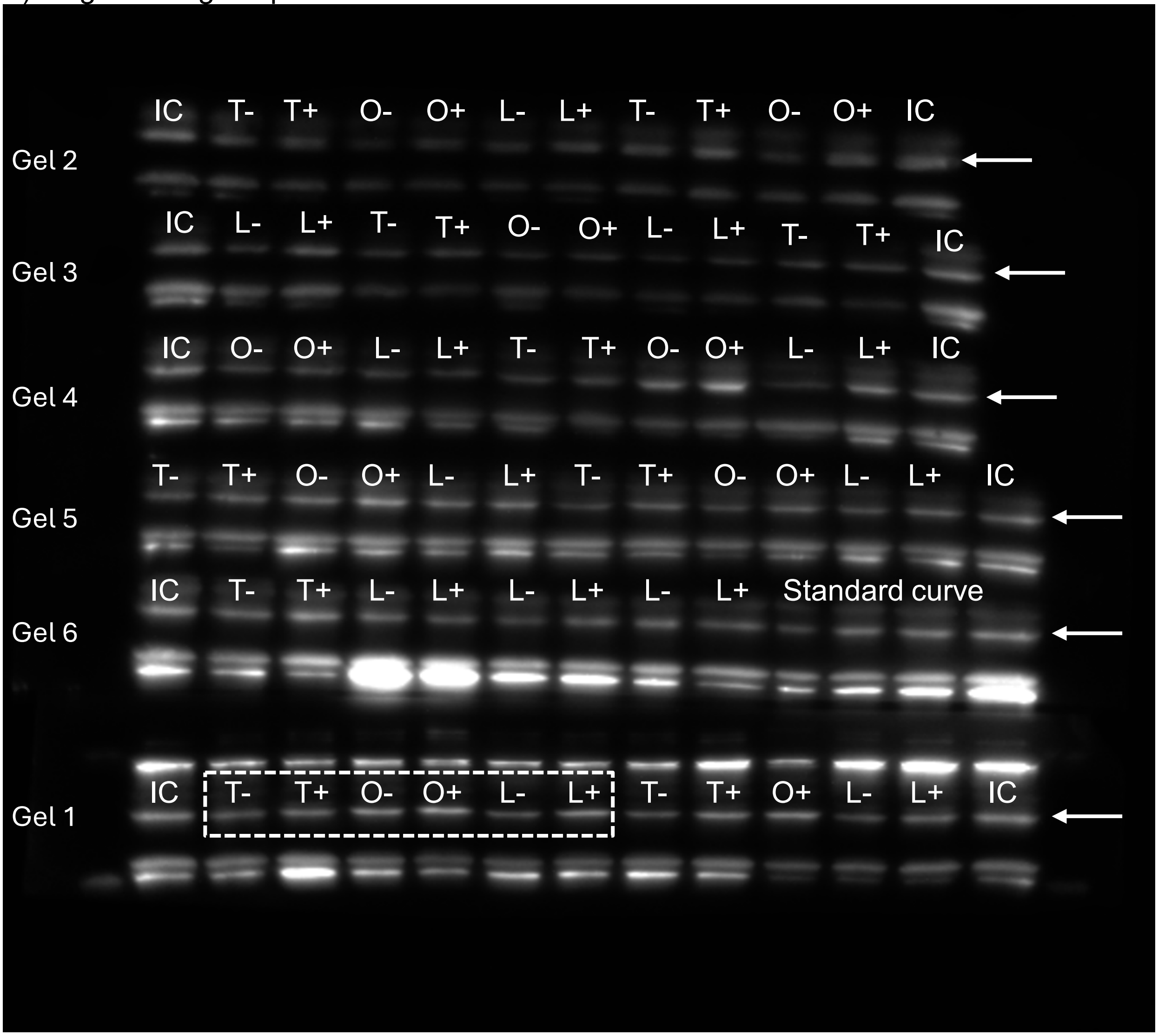

b) Original image inverted using ImageJ for improve visualisation

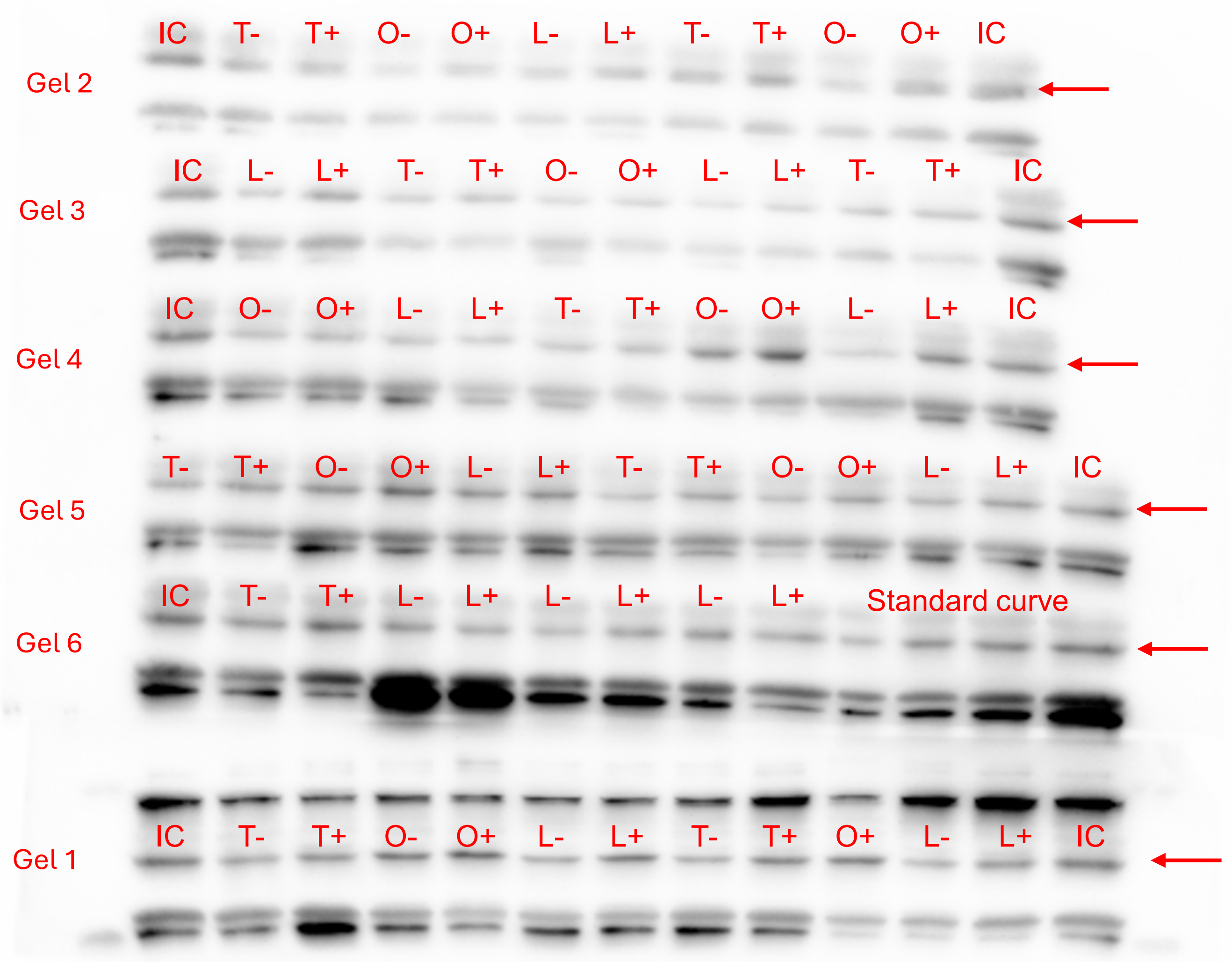

Supplemental figure 4: Immuno blots of XBP-1S (30-50 kDa) in the discovery cohort: a) Original image exported from software and b) original image inverted using ImageJ for improved visualisation. Amount of protein loaded (IC and samples): 4 ug. Amount of protein loaded in the standard curve: 2, 4, 8, and 12 ug. Exposure time: 45 sec.

The nature of the samples are indicated as follows: IC = Internal control, L- = Lean, basal, L+ = Lean, insulin, O- = Obese, basal, O+ = Obese, insulin, T- = T2D, basal, and T+ = T2D, insulin

The samples were run on six gels, which were cut around the band of interest and blotted onto a single membrane to avoid blotting-related differences across the study. The band indicted by the arrow was quantified. A standard curve made from a pooled samples was included on one of the gels as indicated.

a) Original image exported from software

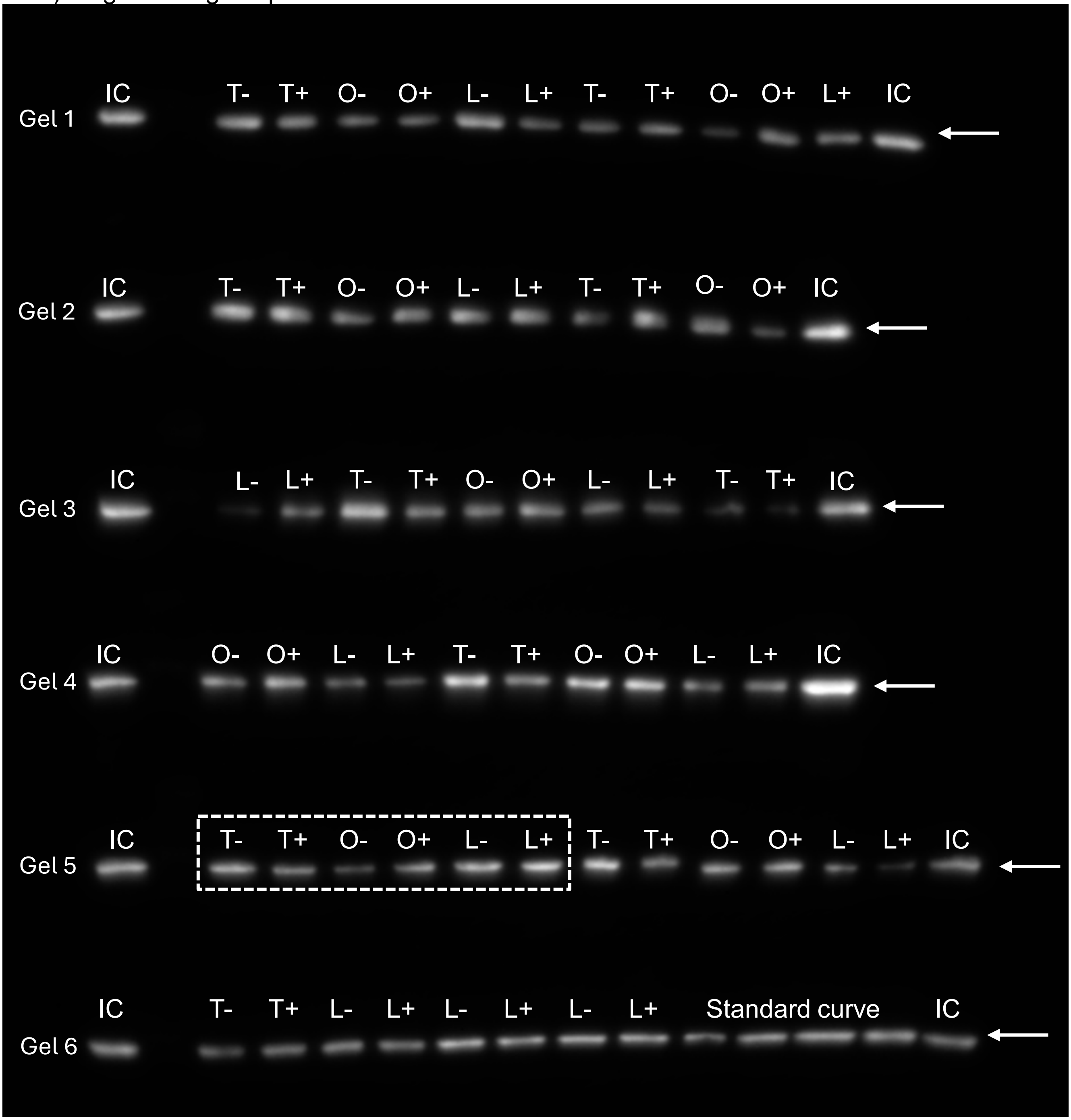

b) Original image inverted using ImageJ for improve visualisation

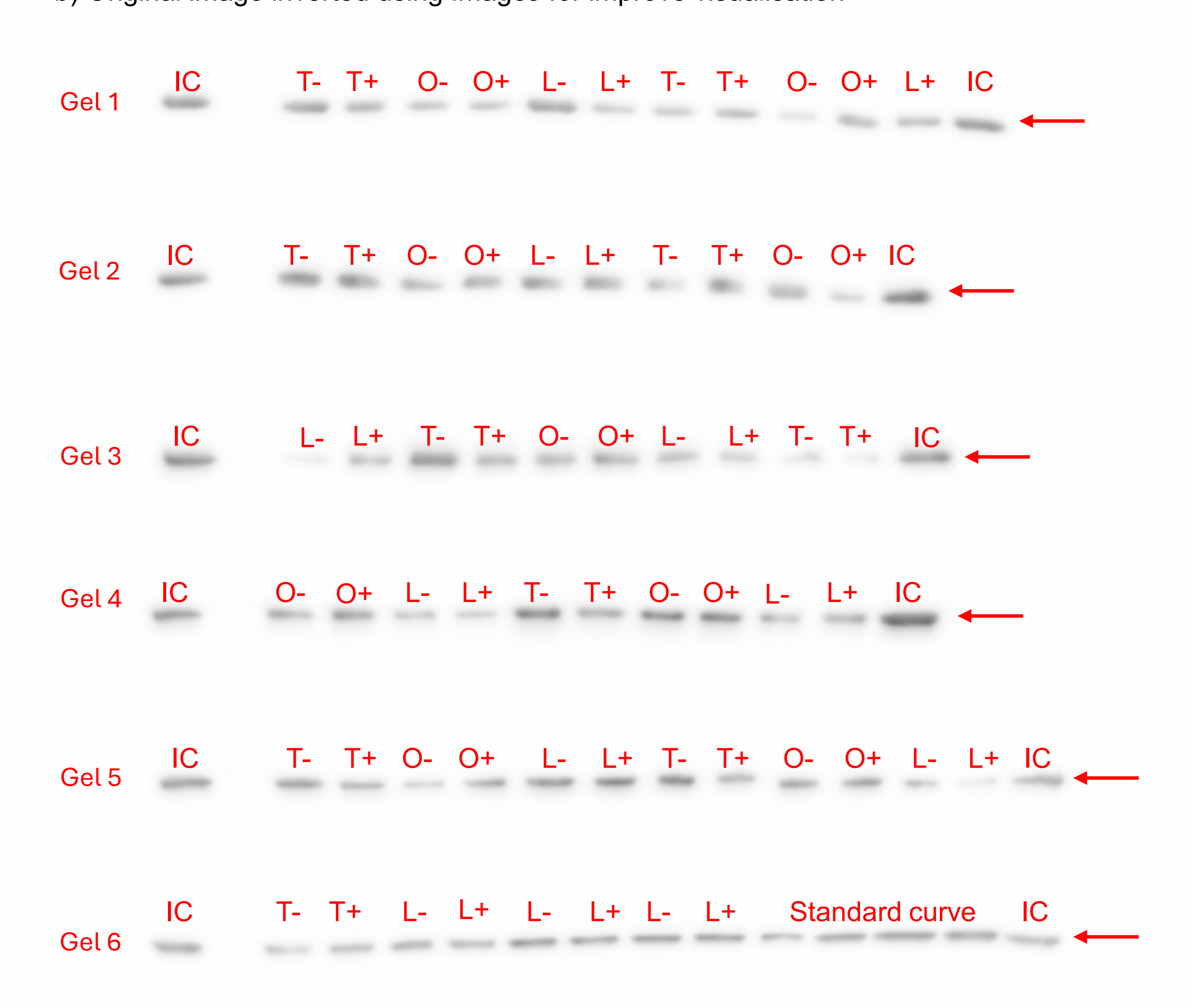

Supplemental figure 5: Immuno blots of XBP-1U (29 kDa) in the discovery cohort: a) Original image exported from software and b) original image inverted using ImageJ for improved visualisation. Amount of protein loaded (IC and samples): 15 ug. Amount of protein loaded in the standard curve: 7,5, 15, 30, and 45ug. Exposure time: 45 sec.

The nature of the samples are indicated as follows: IC = Internal control, L- = Lean, basal, L+ = Lean, insulin, O- = Obese, basal, O+ = Obese, insulin, T- = T2D, basal, and T+ = T2D, insulin

The samples were run on six gels, which were cut around the band of interest and blotted onto a single membrane to avoid blotting-related differences across the study. The band indicted by the red arorow was quantified. A standard curve made from a pooled samples was included on the 6th gel.

a) Original image exported from software

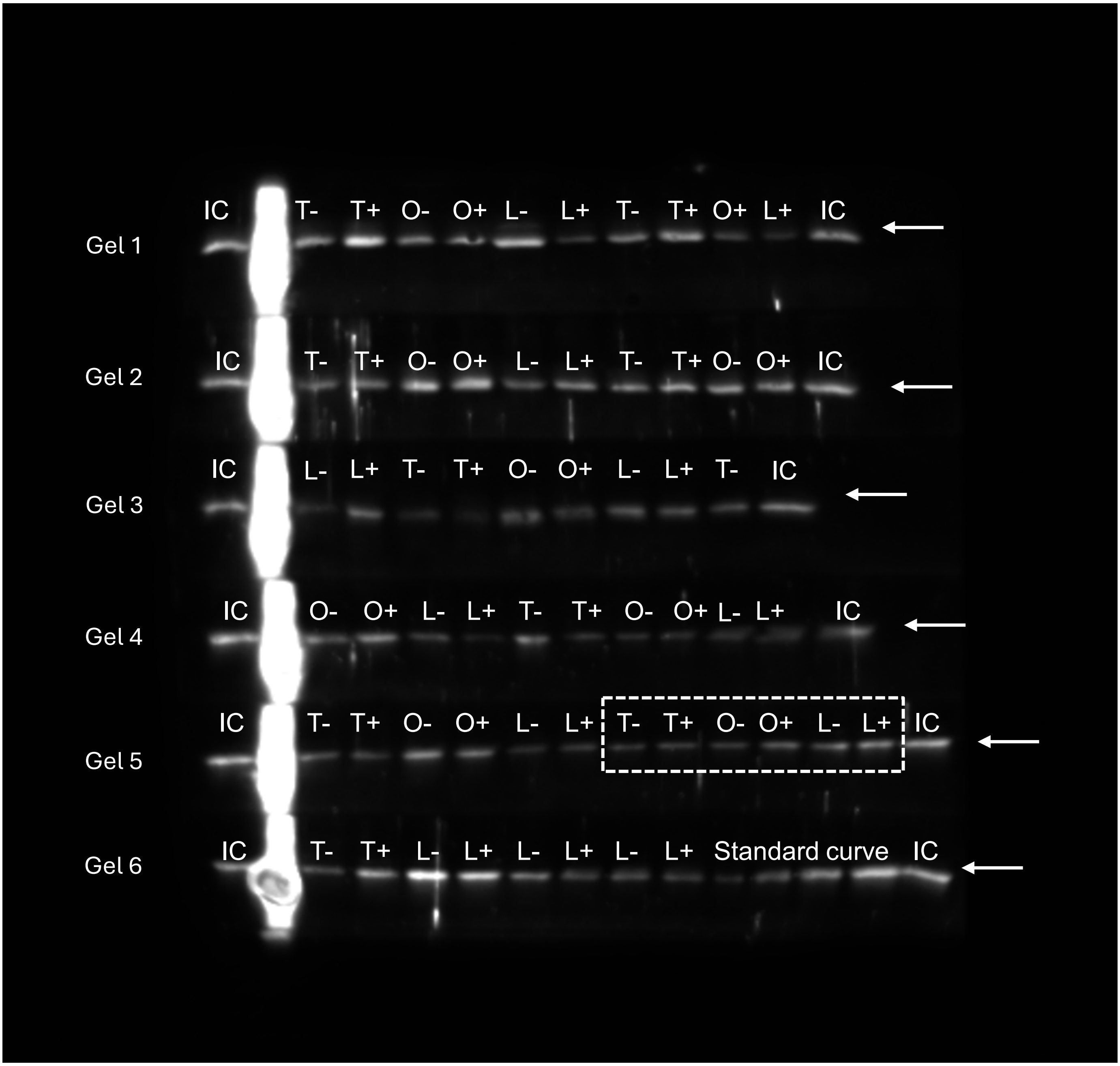

b) Original image inverted using ImageJ for improve visualisation

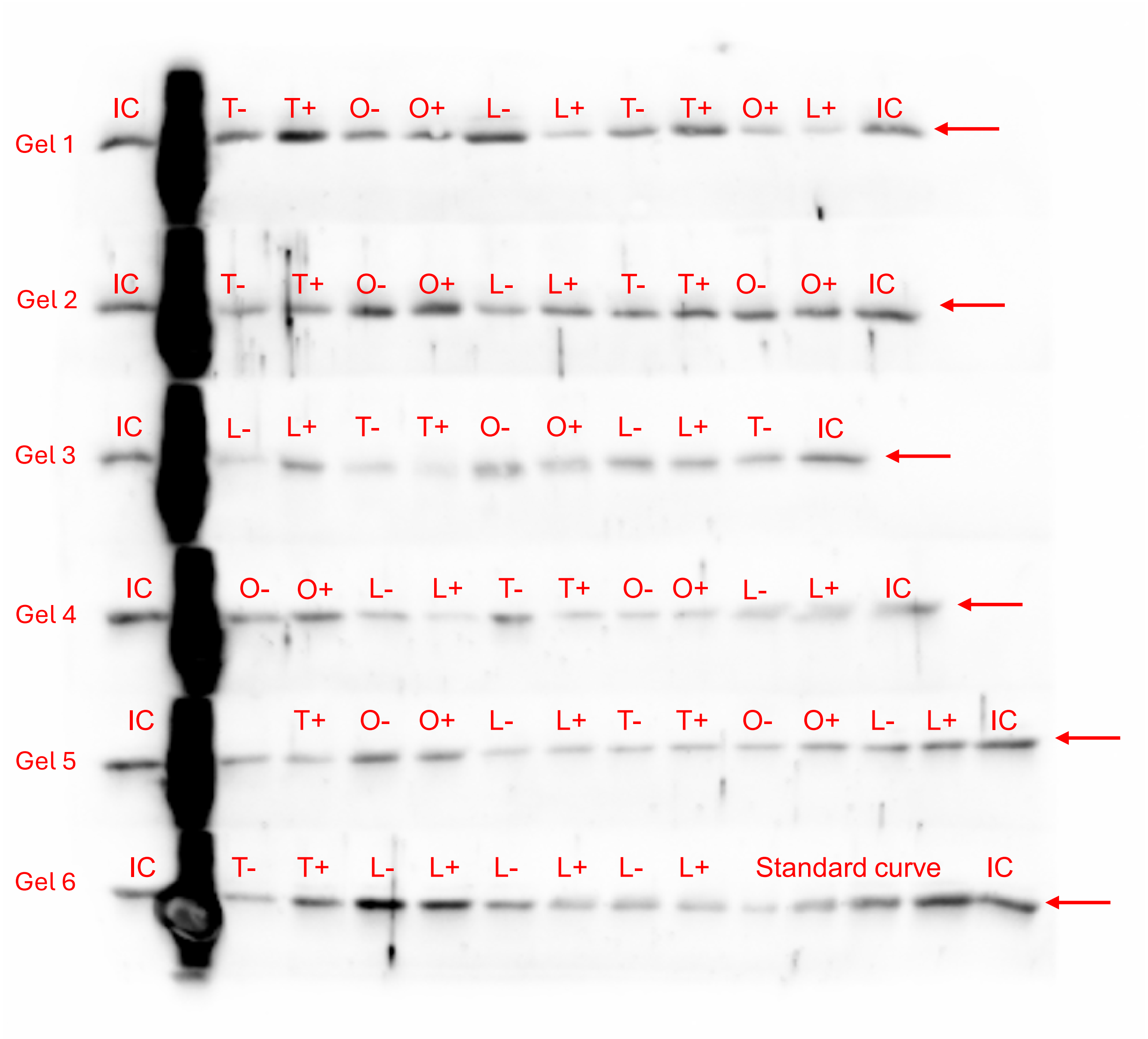

Supplemental figure 6: Immuno blots of GRP78 (78 kDa) and GRP94 (94 kDa) in the discovery cohort: a) Original image exported from software and b) original image inverted using ImageJ for improved visualisation. Amount of protein loaded (IC and samples):30 ug. Amount of protein loaded in the standard curve: 15, 30, 45, and 60 ug. Exposure time: 60 sec.

The nature of the samples are indicated as follows: IC = Internal control, L- = Lean, basal, L+ = Lean, insulin, O- = Obese, basal, O+ = Obese, insulin, T- = T2D, basal, and T+ = T2D, insulin

The samples were run on six gels, which were cut around the band of interest and blotted onto a single membrane to avoid blotting-related differences across the study. The band indicted by the arorow was quantified. A standard curve made from a pooled samples was included on one of the gels as indicated.

a) Original image exported from software

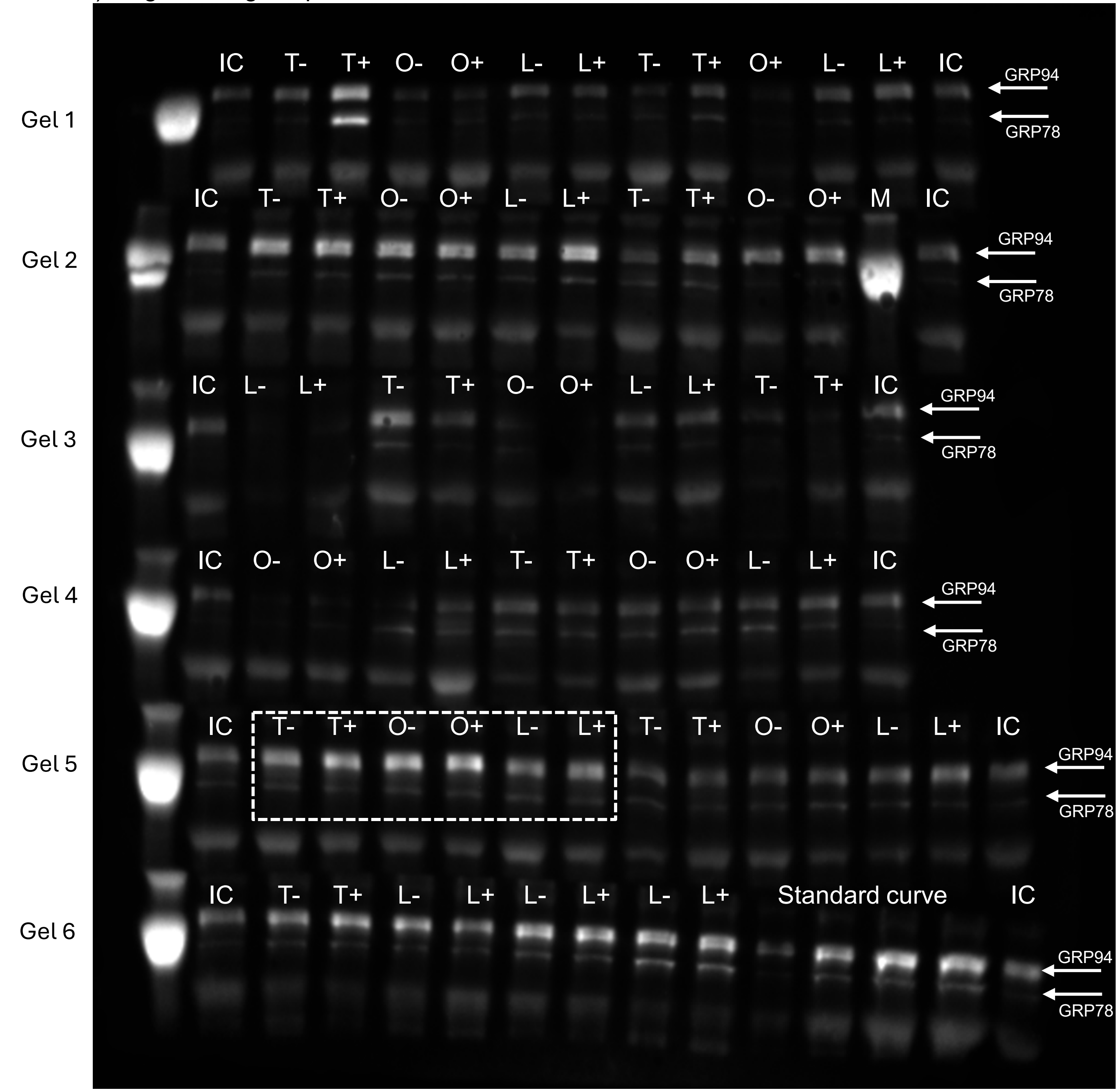

b) Original image inverted using ImageJ for improve visualisation

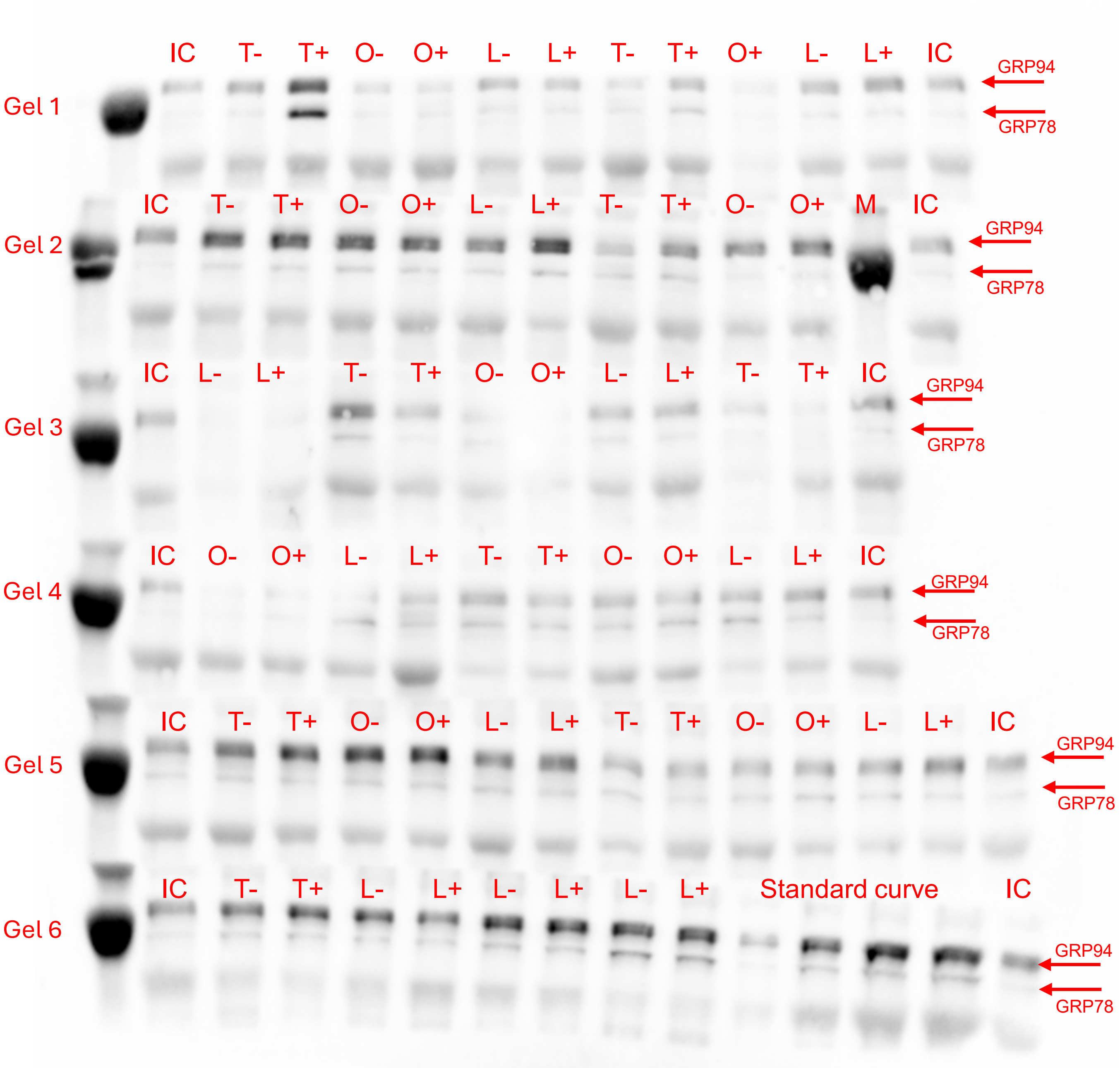

Supplemental figure 7: Immuno blots of PDI (140 kDa) in the discovery cohort: a) Original image exported from software and b) original image inverted using ImageJ for improved visualisation. Amount of protein loaded (IC and samples):30 ug. Amount of protein loaded in the standard curve: 8, 15, 30, and 60 ug. Exposure time: 60 sec.

The nature of the samples are indicated as follows: IC = Internal control, L- = Lean, basal, L+ = Lean, insulin, O- = Obese, basal, O+ = Obese, insulin, T- = T2D, basal, and T+ = T2D, insulin

The samples were run on six gels, which were cut around the band of interest and blotted onto a single membrane to avoid blotting-related differences across the study. The band indicted by the arorow was quantified. A standard curve made from a pooled samples was included on one of the gels as indicated.

a) Original image exported from software

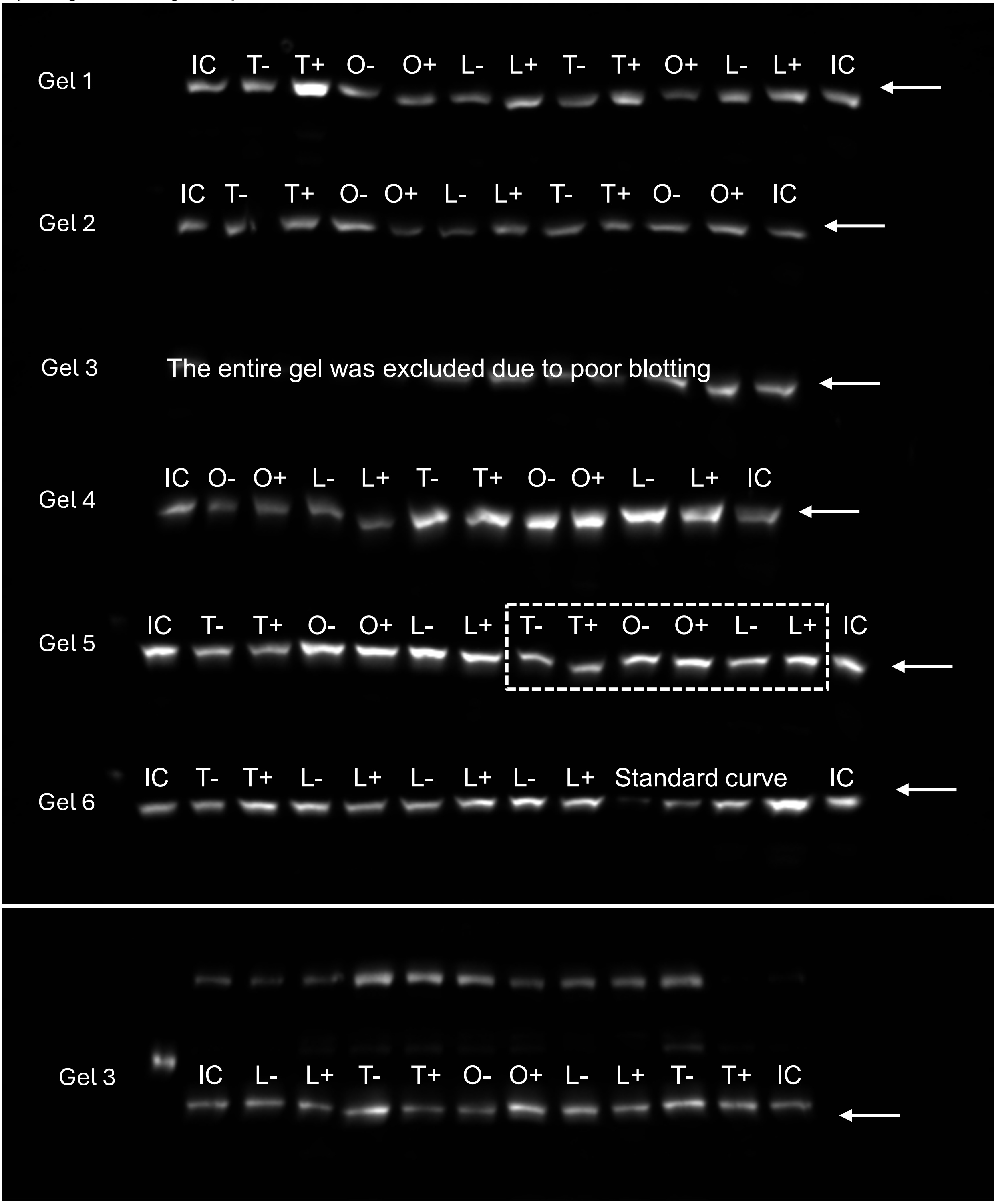

b) Original image inverted using ImageJ for improve visualisation

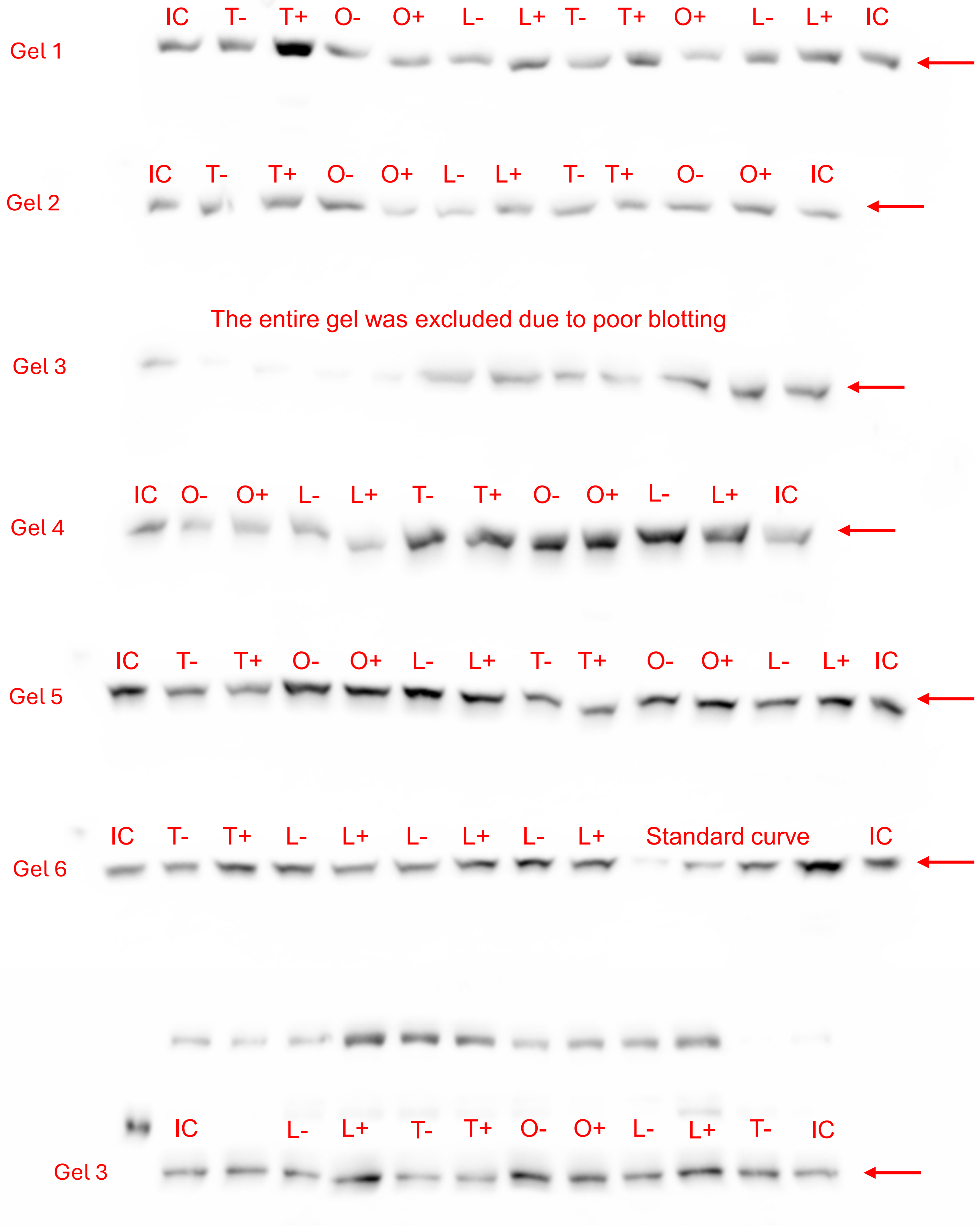

Supplemental figure 8: Immuno blots of CHOP (30 kDa) in the discovery cohort: a) Original image exported from software and b) original image inverted using ImageJ for improved visualisation. Amount of protein loaded (IC and samples): 20 ug. Amount of protein loaded in the standard curve: 10, 20, 30, and 40 ug. Exposure time: 50 sec.

The nature of the samples are indicated as follows: IC = Internal control, L- = Lean, basal, L+ = Lean, insulin, O- = Obese, basal, O+ = Obese, insulin, T- = T2D, basal, and T+ = T2D, insulin

The samples were run on six gels, which were cut around the band of interest and blotted onto a single membrane to avoid blotting-related differences across the study. The band indicted by the arorow was quantified. A standard curve made from a pooled samples was included on one of the gels as indicated.

a) Original image exported from software

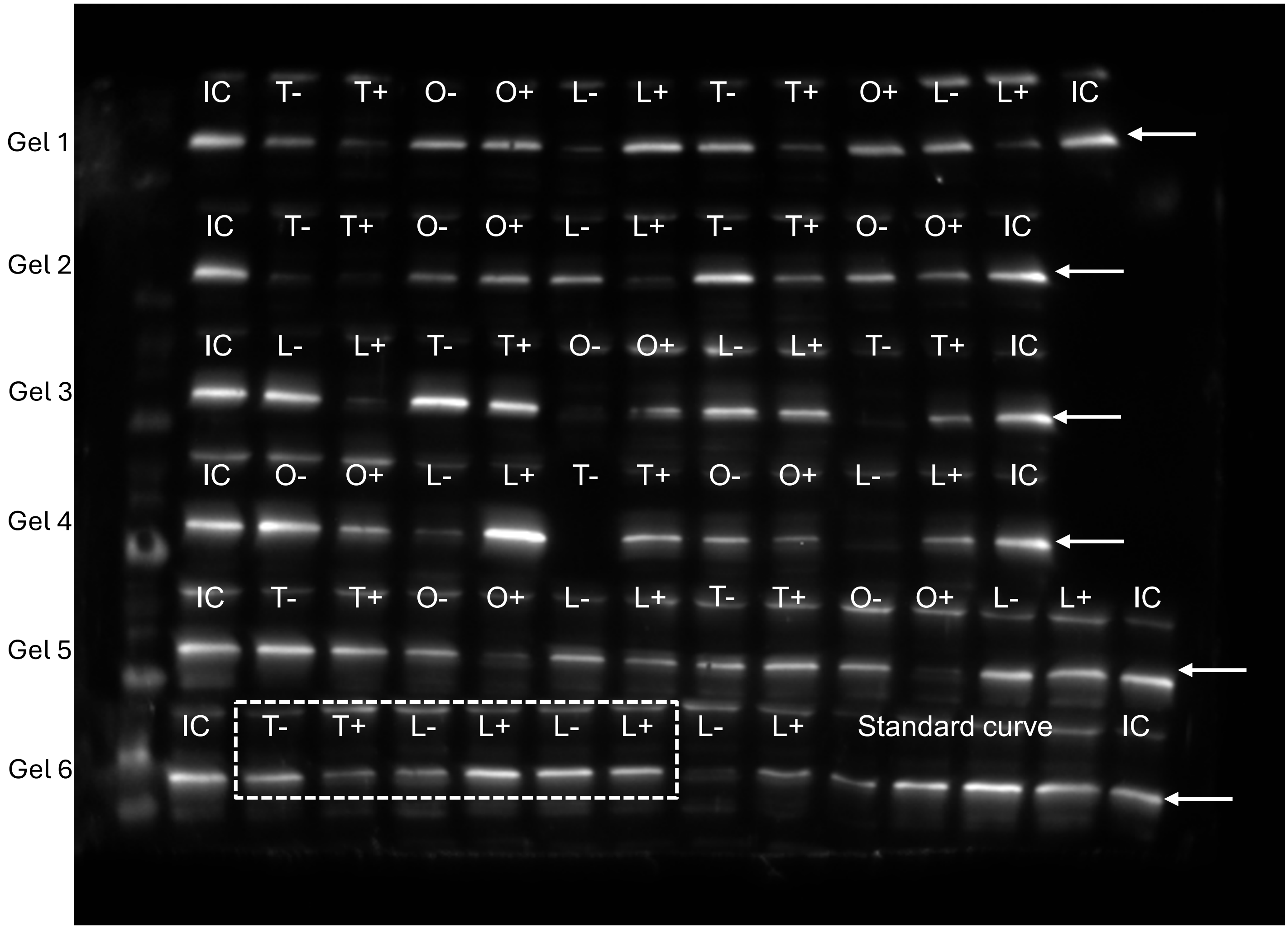

b) Original image inverted using ImageJ for improve visualisation

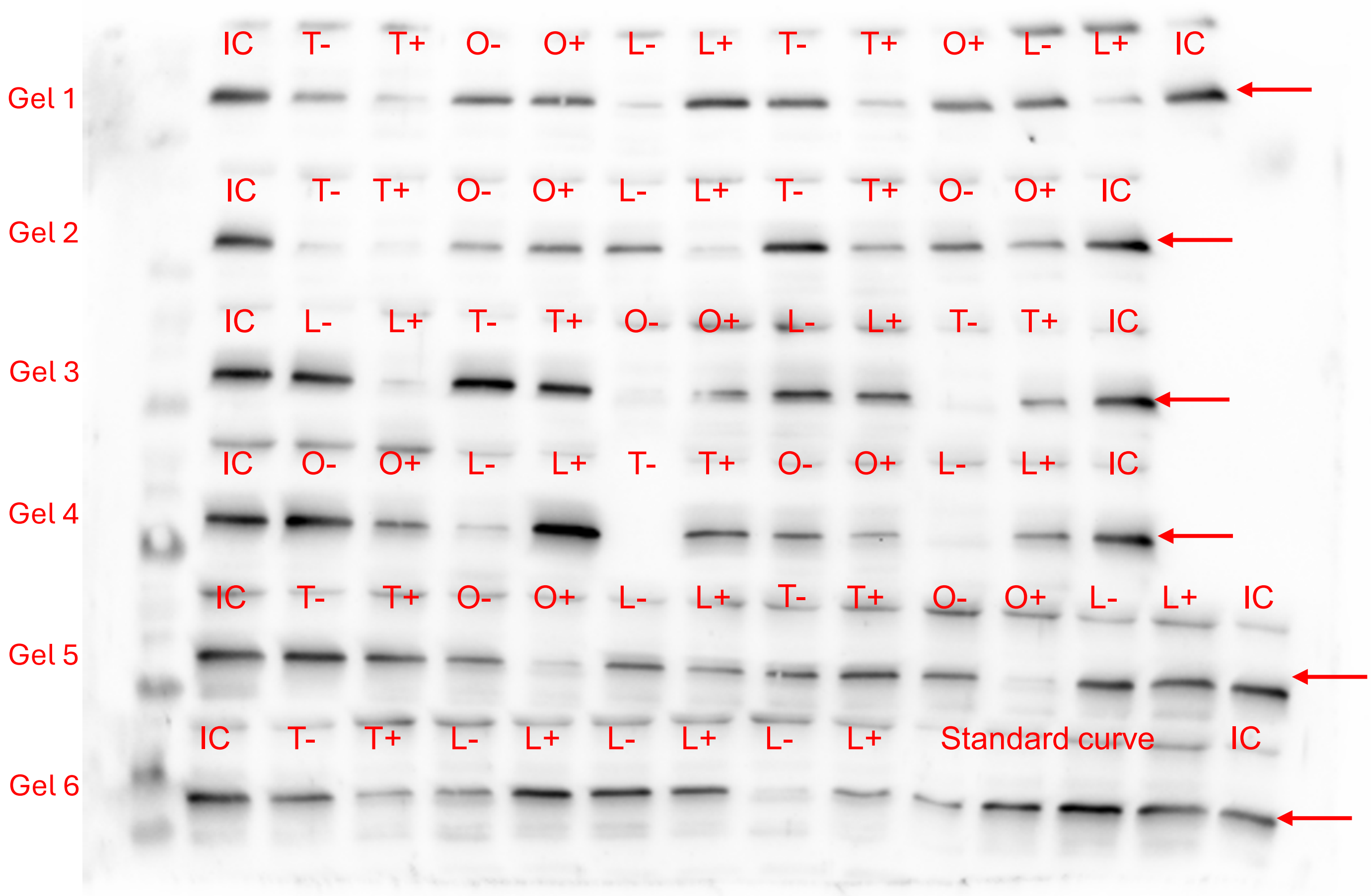

Supplemental figure 9: Immuno blots of ATF3 (30 kDa) in the discovery cohort: a) Original image exported from software and b) original image inverted using ImageJ for improved visualisation. Amount of protein loaded (IC and samples):20 ug. Amount of proteint loaded in the standard curve: 10, 20, 30, and 40 ug. Exposure time: 24 sec.

The nature of the samples are indicated as follows: IC = Internal control, L- = Lean, basal, L+ = Lean, insulin, O- = Obese, basal, O+ = Obese, insulin, T- = T2D, basal, and T+ = T2D, insulin

The samples were run on six gels, which were cut around the band of interest and blotted onto a single membrane to avoid blotting-related differences across the study. The band indicted by the arorow was quantified. A standard curve made from a pooled samples was included on one of the gels as indicated.

a) Original image exported from software

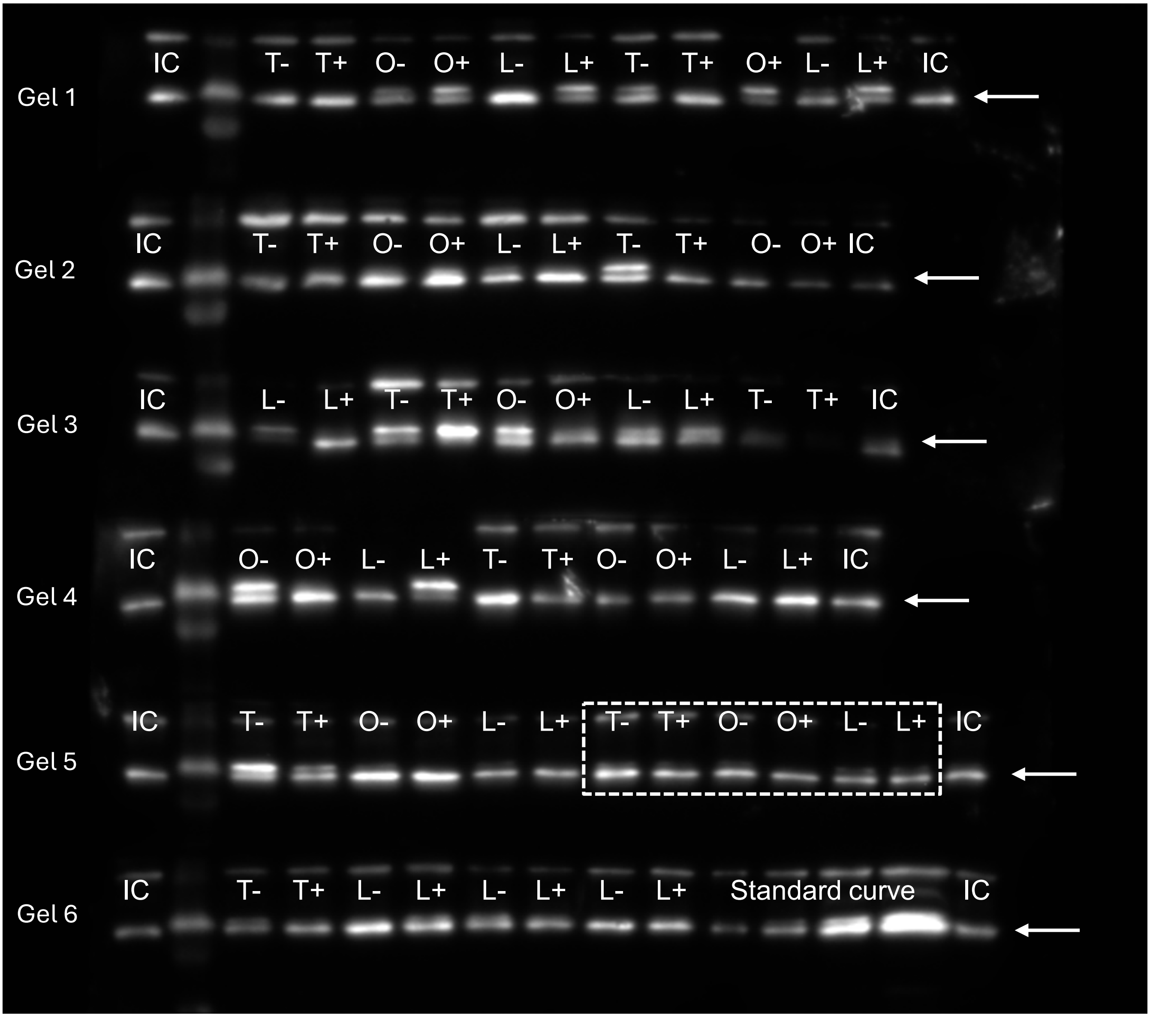

b) Original image inverted using ImageJ for improve visualisation

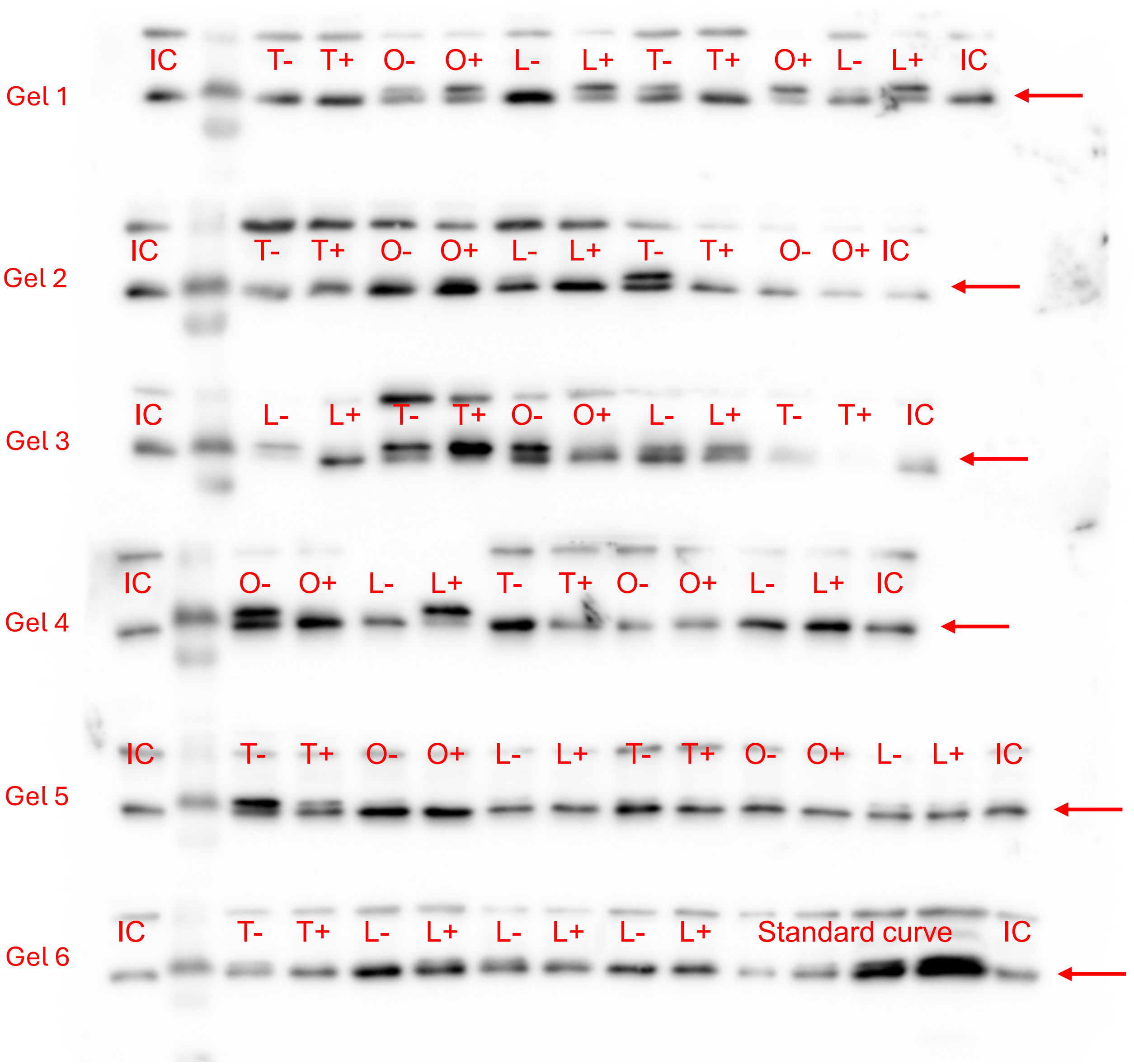

Supplemental figure 10: Immuno blots of MFN2 (84 kDa) in the discovery cohort: a) Original image exported from software and b) original image inverted using ImageJ for improved visualisation. Amount of protein loaded (IC and samples):10 ug. Exposure time: 90 sec.

The nature of the samples are indicated as follows: IC = Internal control, L- = Lean, basal, L+ = Lean, insulin, O- = Obese, basal, O+ = Obese, insulin, T- = T2D, basal, and T+ = T2D, insulin

The samples were run on six gels, which were cut around the band of interest and blotted onto a single membrane to avoid blotting-related differences across the study. The band indicted by the arorow was quantified.

a) Original image exported from software

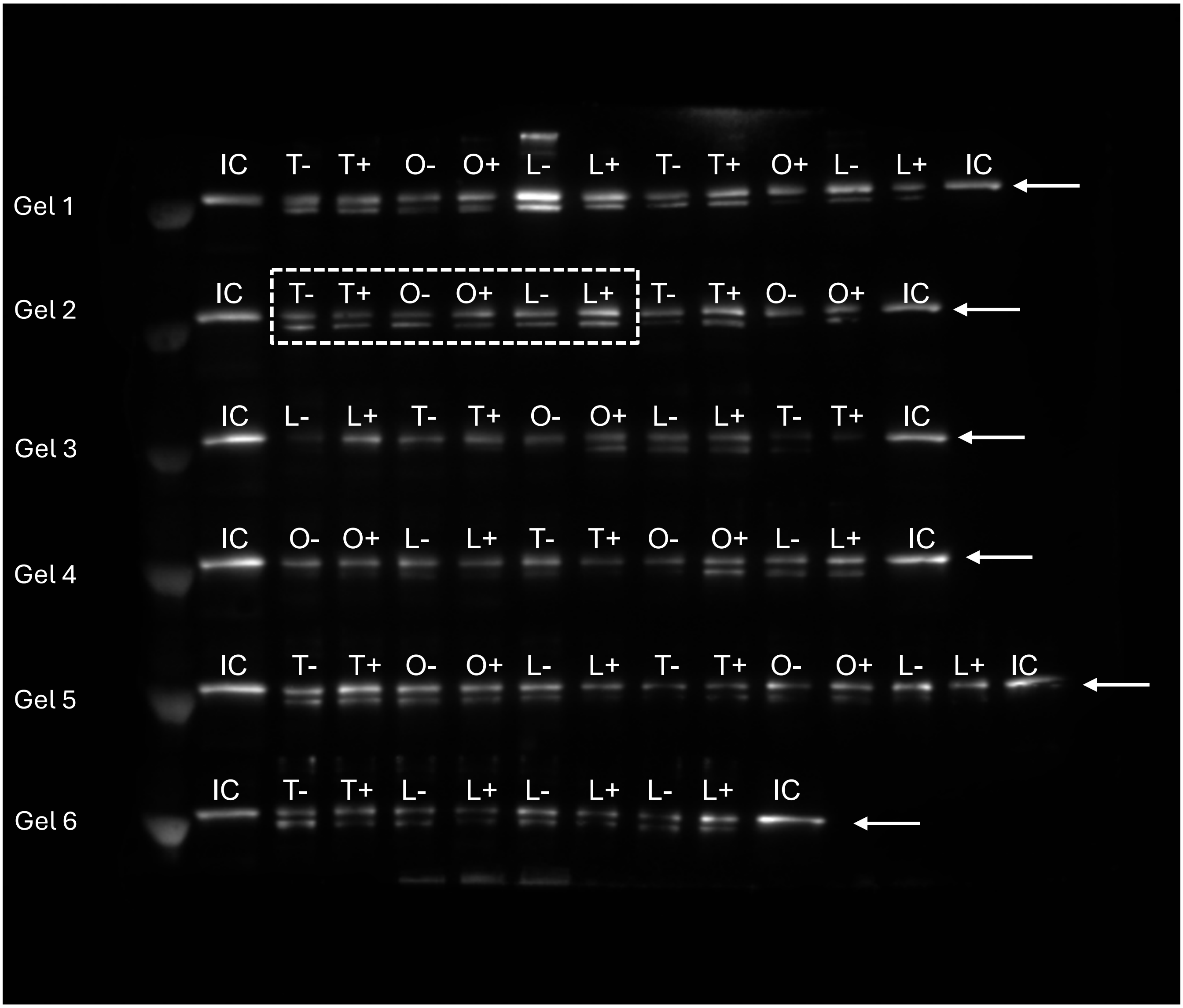

b) Original image inverted using ImageJ for improve visualisation

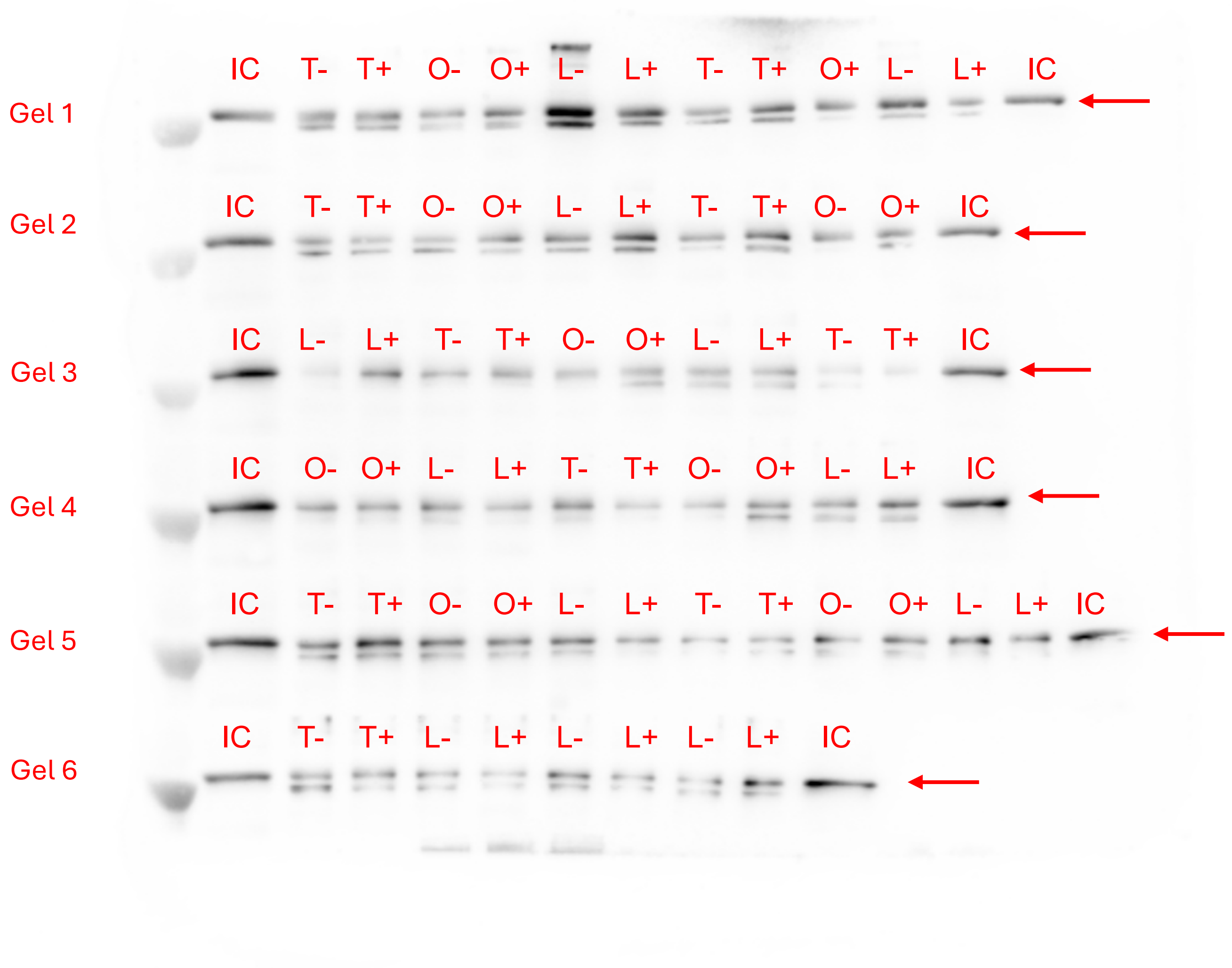

Supplemental figure 11: Immuno blots of eIF2α pSer51(38 kDa) in the validation cohort: a) Gel 1-4 + a gel with a standard curve and b) gel 5-8 + a gel with a standard curve. Amount of protein loaded (IC and samples):7.5 ug. Amount of proteint loaded in the standard curve: 2.5, 5, 7.5, 10, and 12.5 ug. Exposure time: 70 sec (upper membrane) and 80 sec (lower membrane).

The nature of the samples are indicated as follows: IC = Internal control, H- = Healthy, glucose-tolerant, basal, H+ = Healthy, glucose-tolerant, insulin, T- = T2D, basal, and T+ = T2D, insulin

The samples were run on eight gels, which were cut around the band of interest and blotted onto two membranes to minimize blotting-related differences across the study. The band indicted by the arrow was quantified. A standard curve made from a pooled samples was included on the last gel on each of the membranes as indicated.

a) Gel 1-4 + a gel with a standard curve

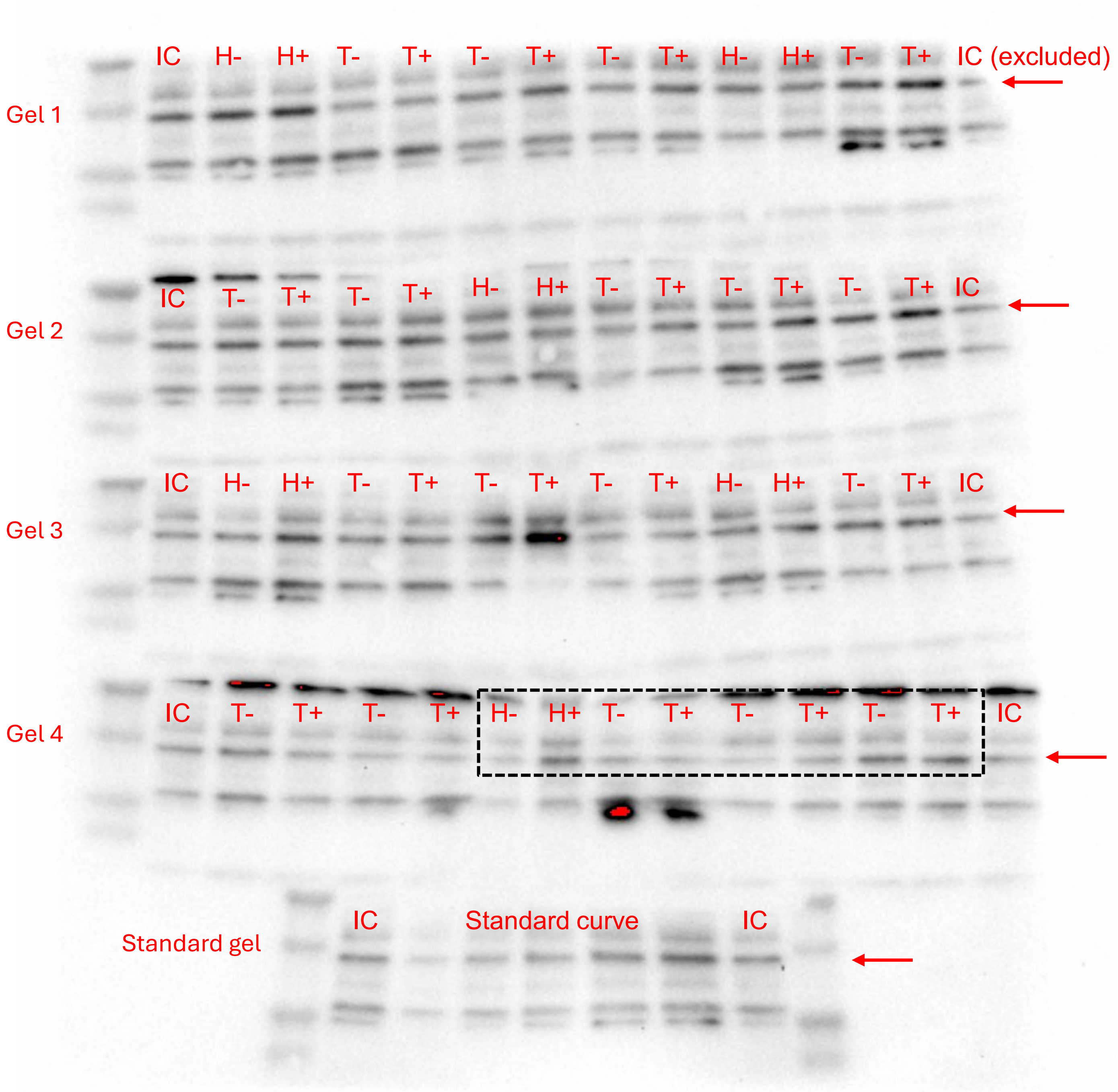

b) Gel 5-8 + a gel with a standard curve

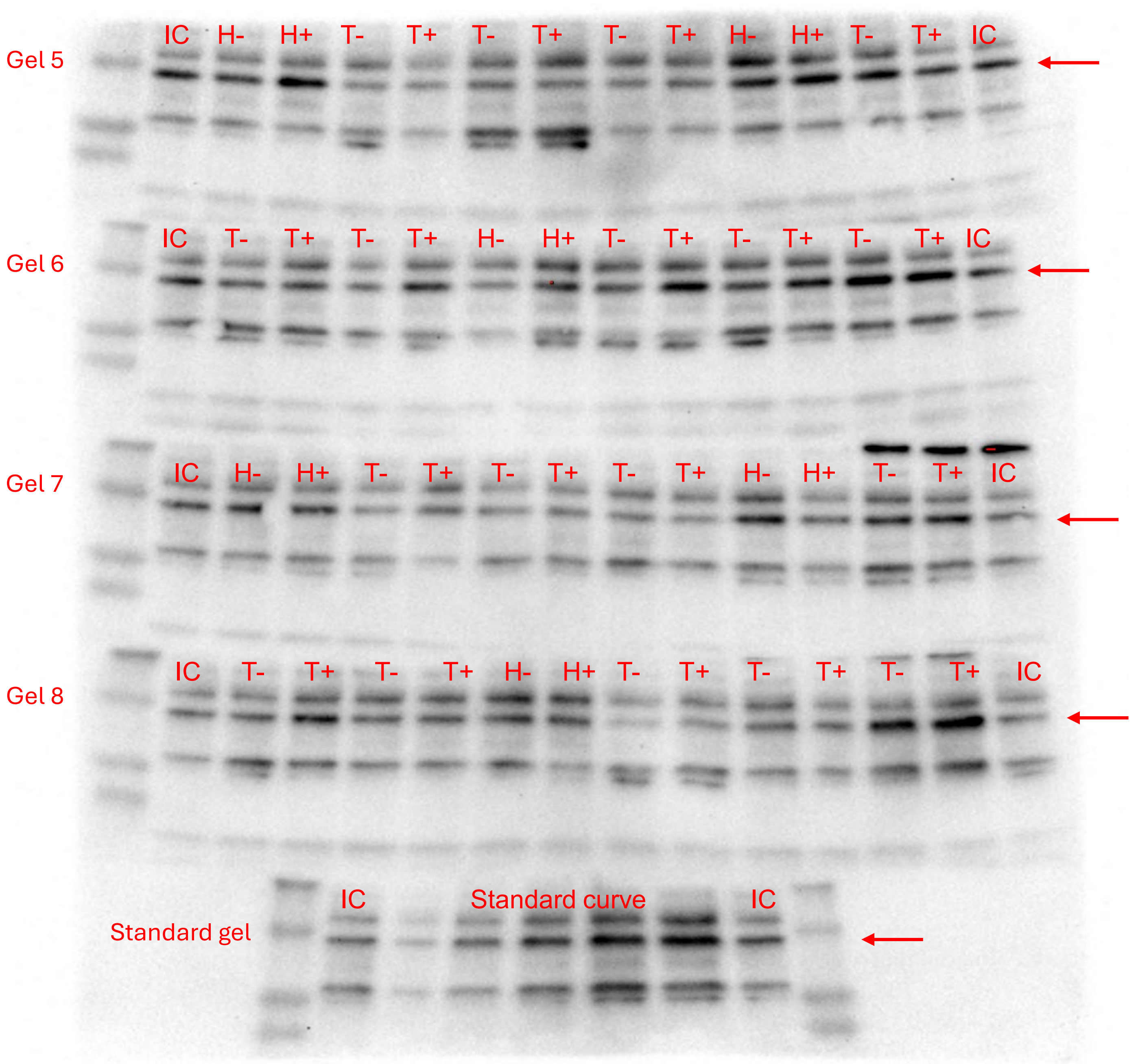

Fig. S12

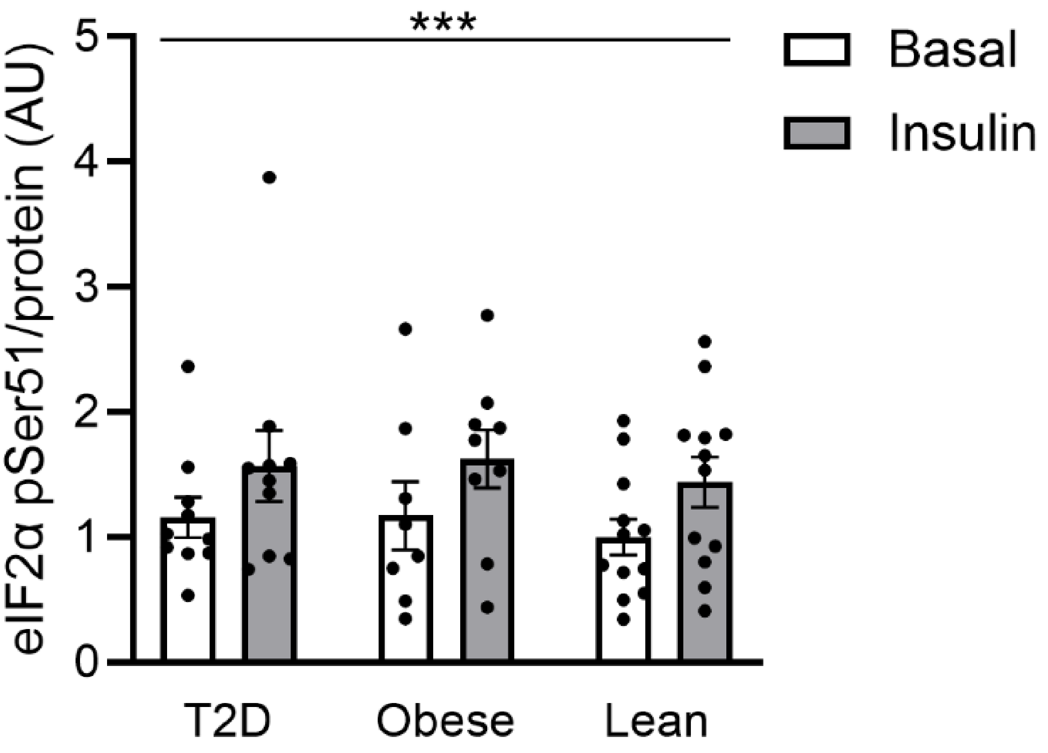

Fig. S12 The eIF2α pSer51/protein ratio in the basal- (white bars) and insulin-stimulated (grey bars) state in skeletal muscle of lean individuals (n=12) and individuals with obesity (n=8-9) as well as in patients with T2D (n=9-10) in the discovery cohort. The data are presented as individual values as well as the mean±SEM. *p<0.05, **p<0.01, and *** p<0.001 vs basal (main effect).
